# Reproduction-associated pathways in females of gibel carp (*Carassius gibelio*) shed light on the molecular mechanisms of the coexistence of asexual and sexual reproduction

**DOI:** 10.1186/s12864-024-10462-4

**Published:** 2024-06-01

**Authors:** Florian Jacques, Tomáš Tichopád, Martin Demko, Vojtěch Bystrý, Kristína Civáňová Křížová, Mária Seifertová, Kristýna Voříšková, Md Mehedi Hasan Fuad, Lukáš Vetešník, Andrea Šimková

**Affiliations:** 1 Department of Botany and Zoology, Faculty of Science, Masaryk University, Kotlářská 2, Brno, 611 37 Czech Republic; 2 Laboratory of Non-Mendelian Evolution, Institute of Animal Physiology and Genetics of the CAS, Liběchov, 277 21 Czech Republic; 3 Faculty of Fisheries and Protection of Waters, University of South Bohemia in České Budějovice, South Bohemian Research Center of Aquaculture and Biodiversity of Hydrocenoses, Zátiší 728/II, Vodňany, 389 25 Czech Republic; 4 Central European Institute of Technology, Masaryk University, Brno, 625 00 Czech Republic; 5 Institute of Vertebrate Biology, Czech Academy of Science, Květná 8, Brno, 603 65 Czech Republic

**Keywords:** Carassius gibelio, Reproduction, Gynogenesis, Asexual reproduction, Evolution of sexual reproduction, Meiosis, Differential expression analysis, Oogenesis, Transcriptomics

## Abstract

Gibel carp (*Carassius gibelio*) is a cyprinid fish that originated in eastern Eurasia and is considered as invasive in European freshwater ecosystems. The populations of gibel carp in Europe are mostly composed of asexually reproducing triploid females (i.e., reproducing by gynogenesis) and sexually reproducing diploid females and males. Although some cases of coexisting sexual and asexual reproductive forms are known in vertebrates, the molecular mechanisms maintaining such coexistence are still in question. Both reproduction modes are supposed to exhibit evolutionary and ecological advantages and disadvantages. To better understand the coexistence of these two reproduction strategies, we performed transcriptome profile analysis of gonad tissues (ovaries) and studied the differentially expressed reproduction-associated genes in sexual and asexual females. We used high-throughput RNA sequencing to generate transcriptomic profiles of gonadal tissues of triploid asexual females and males, diploid sexual males and females of gibel carp, as well as diploid individuals from two closely-related species, *C. auratus* and *Cyprinus carpio*. Using SNP clustering, we showed the close similarity of *C. gibelio* and *C. auratus* with a basal position of *C. carpio* to both *Carassius* species. Using transcriptome profile analyses, we showed that many genes and pathways are involved in both gynogenetic and sexual reproduction in *C. gibelio*; however, we also found that 1500 genes, including 100 genes involved in cell cycle control, meiosis, oogenesis, embryogenesis, fertilization, steroid hormone signaling, and biosynthesis were differently expressed in the ovaries of asexual and sexual females. We suggest that the overall downregulation of reproduction-associated pathways in asexual females, and their maintenance in sexual ones, allows the populations of *C. gibelio* to combine the evolutionary and ecological advantages of the two reproductive strategies. However, we showed that many sexual-reproduction-related genes are maintained and expressed in asexual females, suggesting that gynogenetic gibel carp retains the genetic toolkits for meiosis and sexual reproduction. These findings shed new light on the evolution of this asexual and sexual complex.

**Supplementary Information:**

The online version contains supplementary material available at 10.1186/s12864-024-10462-4.

## Introduction

The establishment of sexual reproduction has been a major event in the evolution of eukaryotes [1]. However, asexual reproduction has evolved independently in dozens of eukaryotic lineages, and is documented in approximately 80 vertebrate species, all representing reptiles, amphibians [2], and teleost fish [3]. Asexual species often originate from hybridization events and/or ploidy alteration [4–7]. These processes usually affect meiosis and generate new species with asexual females only [8–11]. Both sexual and asexual reproduction exhibit various evolutionary and ecological advantages and disadvantages. The main disadvantage of sexual reproduction is the two-fold cost of meiosis and the production of male offspring [12]. Consequently, sexual individuals can be outnumbered by parthenogenetic females that exhibit twice the egg production rate. On the other hand, parthenogenetic forms suffer from the accumulation of deleterious mutations and reduced adaptive abilities, including lower ecological tolerance and higher susceptibility to parasites, following the principle of Muller`s ratchet [13]. Hence, asexually reproducing species are usually considered a short-term evolutionary dead-end, and this explains the maintenance of sexual reproduction in the vast majority of eukaryotic lineages [14]. Still, asexual reproduction persists in nature, and for some vertebrates, sexual and asexual complexes of closely-related species often coexist with sexual forms in the same habitats (e.g., the teleosts *Poecilia* and *Cobitis*, and the lizard *Aspidoscelis*) [6, 15, 16].

Interspecific hybridization played an important role in the formation of polyploid asexual species. In amphibians and teleosts, all-female asexual species reproduce by gynogenesis [17], a process where females use the sperm from males of the same species or a closely-related species to induce embryogenesis, without the contribution of paternal genetic material to the offspring. Regarding fish, several asexual-sexual complexes have been reported. The asexual North American leuciscid *Phoxinus eos-neogaeus* is the result of interspecific hybridization between the sexual species *P. eos* and *P. neogaeus* [18]. In the European *Cobitis* complex, hybridization between sexual species generated sterile males and asexual triploid females that produce eggs through premeiotic endoreplication [6, 19]. The asexual *Poecilia formosa* from the Amazon basin, which forms eggs through achiasmatic meiosis without recombination [15], results from hybridization between two sexual species, *P. mexicana* and *P. latipinna* [20]. The Iberian minnow *Leuciscus alburnoides* represents another case of a species resulting from hybridization, this with a complex genetic constitution and exhibiting the coexistence of diploid and triploid forms, as well as gynogenesis and sexual reproduction [21, 22].

The gibel carp (*Carassius gibelio*), also known as Prussian carp, considered as a member of the *C. auratus* complex or with a species status [23, 24], is a cyprinid fish originating from eastern Eurasia that became invasive in European freshwater ecosystems during the 20th century, due to its high ecological tolerance and adaptive abilities [25, 26]. Gibel carp exhibits a dual mode of reproduction - sexual reproduction and gynogenesis [23, 27–29]. The emergence of asexual reproduction in this species is concomitant with a triploidization event [30]. The first populations invading the freshwaters of the Czech Republic around 1975 [31] included only triploid asexual females. Fifteen years later, mixed populations composed of triploid asexual females and diploid females and males reproducing sexually appeared. A low proportion of triploid and tetraploid males was also reported [31, 32]. In Asian populations of *C. auratus gibelio* (following the taxonomy used by Asian authors), this phenomenon was explained by allogynogenesis, where heterologous sperm sometimes contribute to the phenotype of the offspring [33]. Zhou et al. (2000) [32] even reported molecular evidence of sexual reproduction in the asexual females of Chinese populations of *C. auratus gibelio*. They suggest that homologous sperm insemination of the eggs of asexual females is similar to classical sexual reproduction (the fused nucleus of the zygote undergoes recombination and removes extra maternal chromosomes). However, there is no empirical evidence of the capacity of sexual reproduction in the asexual form of *C. gibelio* distributed across Europe.

The coexistence of the two reproduction forms in *C. gibelio* might be a unique case of the switch from a unisexual species to a partly sexual species. Several mechanisms have been proposed to explain the coexistence of asexual and sexual individuals (summarized by Knytl et al. [23]). The Red Queen hypothesis predicts evolution towards equilibrium in the populations of sexual and asexual forms coexisting together and co-evolving with parasites. As asexual reproduction is associated with reduced genetic diversity, parasitism is supposed to play an important role in the maintenance of sexual reproduction [28, 34]. Clonally reproducing females of *C. gibelio* suffer from higher parasite loads when compared to the genetically variable sexual form which is expected to escape the parasite infection. Sexual selection also increases the variability of immune genes, therefore sexual diploids show higher genetic diversity in immune genes than asexual triploids, in accordance with the Red Queen hypothesis [28]. The coexistence of the two reproduction forms in fish may also be facilitated by other ecological processes, such as male discrimination against asexual females [35], the generation of sexual individuals from asexual females [36], the differential competitive abilities of asexuals and sexuals [37], and the occupation of different ecological niches [38]. While asexual reproduction allows for a quick clonal multiplication of individuals in stable environments [39], sexual reproduction favors genetic diversity [40], heterozygosity, and DNA repair, and hence adaptation to changing environments. Moreover, the necessity of asexual forms to coexist with sexual forms is directly related to gynogenesis, which requires males of conspecifics or close species in the same habitats for egg activation.

*Carassius gibelio* represents a unique example of a species where sexual and asexual forms coexist [34]. Hence, this species constitutes an object of study to elucidate the evolution of sexuality and asexuality in animals, and the mechanisms responsible for the stable coexistence of sexual and asexual individuals. Furthermore, the origin of *C. gibelio* is still in question. The widely accepted hypothesis is that *C. gibelio* arose from autotriploidization within the evolutionary branch of the *C. auratus* complex, leading to triploid asexual females [41–44]. However, Yuan et al. (2010) [45], focusing on hox genes, suggested the potential hybrid origin of triploid asexual *C. gibelio* from *C. auratus* and *C. carpio*. Understanding the role of polyploidization in the origin of *C. gibelio*, and the extent of the genomic contribution of *C. carpio* and *C. auratus* to *C. gibelio*, could provide a better understanding of the evolution of asexual and sexual reproduction in *C. gibelio*.

Here, the molecular mechanisms associated with reproduction in *C. gibelio* were analysed to study the coexistence of asexual and sexual forms. In particular, the expression of reproduction-related genes was expected to differ between asexual and sexual females, since meiosis-related genes are not important for asexually reproducing individuals. To test this hypothesis, transcriptome profile analyses of gonadal tissues (ovaries) from asexual females and sexual females of *C. gibelio* were performed. In addition, the transcriptomes of the closely-associated species *C. carpio* and *C. auratus* were also analysed, with a particular emphasis on the genes contributing to sexual reproduction.

## Materials and methods

### Fish tissue sampling

Asexual and sexual *C. gibelio* were obtained from artificial breeding of the parental fish collected in their natural habitats. Parental *C. gibelio* were sampled in the locality (Dyje River, Czech Republic) and genotyped following the approach of Pakosta et al. [46], Papoušek et al. [47] and Šimková et al. [48]. Asexual female offspring was obtained by induced embryogenesis using sperm of *C. carpio*. The sexual offspring was obtained from the artificial interbreeding of sexual specimens. The ploidy of parental asexual females used for gynogenesis (i.e. induced embryogenesis by *C. carpio*) and parental sexual specimens used for interbreeding was analysed by flow cytometry following Šimková et al. (2015). From each fish, fin clip about 1 cm^2^ was sampled for ploidy detection. Before analysis this tissue was homogenised with scissors on Petri dish in 2 ml solution of CyStain DNA 1 step PARTEC and relative DNA content was estimated using Partec CCA I flow cytometer (Partec GmbH; www.sysmex-par tec.com). Diploid *C. auratus* was used as a reference standard. All parental *C. gibelio* were also genotyped for mtDNA (D-loop) and microsatellite loci were amplified following Papoušek et al. (2008), Šimková et al. (2013) and Pakosta et al. (2018).

The fish offspring was reared in aquarium conditions until the age of four years and subsequently their gonadal tissues were sampled (the age of the examined fish corresponded to 4+). *Cyprinus carpio* and *Carassius auratus* were obtained from external breeding facilities. Fish were euthanized using physical stunning through a blow to the skull with a blunt wooden instrument immediately followed by exsanguination.

Four or five biological samples per fish group from a total of 8 fish groups were analysed (females and males of *C. gibelio* resulting from sexual reproduction, females and temperature-induced males of *C. gibelio* resulting from gynogenesis, females and males of sexual *C. auratus*, and females and males of sexual *C. carpio*). Gonadal tissues of each fish specimen were individually submerged in Ambion RNAlater stabilization solution (Thermo Fisher Scientific, Waltham, MA, USA). Tubes with tissues were stored at -80ºC until the isolation of total RNA. Prior to sampling, ploidy of each *C. gibelio* specimen was checked using the same methodology as described above.

### RNA extraction and library preparation

Total RNA was isolated from the gonad tissue of each fish specimen. For extraction, PureLink® RNA Mini Kit (Ambion) with Trizol reagent (Thermo Fisher Scientific) and on-column PureLink DNase treatment were used according to the manufacturer´s protocol. Reagent and buffer volumes were adjusted according to the weight of tissue entering the isolation process (30 mg on average). The final elution was performed using 100 µl of RNAse-free water in the first step and the primal eluate in the second step. The yield and concentration of RNA isolates were checked using a QubitTM 4 fluorometer (Invitrogen by Thermo Fisher Scientific) and Qubit RNA HS Assay Kit (Thermo Fisher Scientific). The quality and integrity of RNA were analysed using RNA 6000 Nano Kit on a 2100 Bioanalyser instrument (Agilent Technologies, Santa Clara, CA, USA). All RNA isolates were normalized by dilution at a uniform concentration of 20 ng/µl with RNase-free water. They served as templates for DNA library preparation in twice the reaction volume recommended by the manufacturer.

All samples (RNA integrity number – RIN > 7) were used for DNA library preparation. 500ng of total RNA was used for mRNA enrichment using the Poly(A) mRNA Magnetic Isolation Module (New England Biolabs, Ipswich, MA, USA). Subsequently, NEBNext® Ultra™ Directional RNA Library Prep Kit for Illumina®, and NEBNext® Multiplex Oligos for Illumina® (Dual Index Primers Set 2, New England Biolabs) were used for library preparation, with 11 PCR cycles utilized for PCR enrichment. RNA fragmentation (13 min at 94 °C) and the size selection conditions (a bead volume of 30 µl and 15 µl for the first and second bead selections, respectively) were further modified in the protocol. The quantification of DNA libraries was performed on a QubitTM 4 fluorometer (Invitrogen by Thermo Fisher Scientific) using Qubit dsDNA HS Assay Kit, and quality and size control were performed on a 2100 Bioanalyser with DNA 1000 Kit (Agilent Technologies). Finally, amplicons were pooled in equimolar amounts. The final concentration of each library in the pool was 10 nM in the pool. Subsequently, NEBNext® Ultra™ Directional RNA Library Prep Kit for Illumina® and NEBNext® Multiplex Oligos for Illumina® (Dual Index Primers Set 2, New England Biolabs) together with spike-in RNA were used for cDNA library preparation from total RNA. The quality of prepared cDNA libraries was evaluated using a Qubit fluorometer (Thermo Fisher Scientific). The quality of cDNA libraries was visualized by a 2100 Bioanalyser (Agilent Technologies), and the libraries were finally sequenced by Macrogen Korea on Illumina HiSeq X (one lane) in a paired-end configuration producing 150 bp long reads. Quality and quantity control steps were carried out by a service company.

### NGS data analyses

A quality check of raw paired-end fastq reads was carried out by FastQC [49] and their origin was categorized using BioBloomTools v2.3.4 [15]. The Illumina adapters clipping and quality trimming of raw fastq reads were performed using Trimmomatic v0.39 [50] with settings CROP:250 LEADING:3 TRAILING:3 SLIDINGWINDOW:4:5 MINLEN:35. Trimmed RNAseq reads were mapped to the *Carassius auratus* genome (ASM336829v1) with Ensembl annotation (release 104) using STAR v2.7.3a [51] as a splice-aware short read aligner and default parameters except for --outFilterMismatchNoverLmax 0.1 and --twopassMode Basic. Quality control after alignment concerning the number and percentage of uniquely- and multi-mapped reads, rRNA contamination, mapped regions, read coverage distribution, strand specificity, gene biotypes, and PCR duplication was performed using several tools – namely, RSeQC v4.0.0 [52], Picard toolkit v2.25.6 [53], and Qualimap v.2.2.2 [54]. All statistics were processed by MultiQC v1.10.1 [55].

### SNP clustering analysis

The genomic sequences of all collected samples were aligned to the *Carassius auratus* reference genome (ASM336829v1-104) utilizing the Burrows-Wheeler Aligner (BWA) software [56]. Post alignment and germline variants were called using Strelka2 variant calling software [57], generating variant calls in VCF format which were further filtered to retain only high-confidence variants. These variants were then annotated using the reference Gene Transfer Format (GTF) file for *Carassius auratus* (ASM336829v1-104). Subsequent data processing was carried out in R, where the variant tables were further refined and merged with sample information. A series of filtering steps were performed to ensure only variants with sufficient coverage and sample counts were retained for analysis. The filtered variant table was then reorganized and formatted for subsequent comparative analyses. Variants located on sex chromosomes were excluded for certain analyses to ensure accurate cross-species comparisons. The data were then restructured to compare SNP identity across species, generating similarity matrices and Venn diagrams to visualize the overlap of SNPs by species and ploidy levels.

### Differential expression analysis and pathway enrichment analysis

Appropriate bioinformatics tools were used for the processing of raw sequencing data. The genome of *C. auratus* was used as reference. The differential gene expression was calculated on the basis of the gene counts produced using featureCounts from the Subread package v2.0 [58] and further analysed by Bioconductor package DESeq2 v1.34.0 [59]. Data generated by DESeq2 with independent filtering were selected for differential gene expression analysis to avoid potential false positive results. Differences in gene expression were considered significant on the basis of a cut-off of the adjusted p-value ≤ 0.05. GO term enrichment was analysed using David [60] to retrieve Gene Ontology terms in the Biological process, Cellular Component and Molecular function categories, as well as KEGG pathways [61, 62]. Graphical representations of the GO enrichment were realized using R [63] and Revigo [64]. Reproduction-associated candidate genes were retrieved using the BlastKoala tool of KEGG [61], the BioMart tool of Ensembl [65], and published studies [20, 66–68]. GO terms enrichment was tested using Fisher’s exact test (α = 0.05) with false discovery rate (FDR) correction of the p-value. To interpret the biological functions of the DEGs, their mapping to the Gene Ontology (GO) [62] and KEGG [61] databases was performed to analyse pathway enrichment. In each of six fish groups associated with sexual reproduction and asexual males, significantly differently-expressed genes (DEGs) compared to the triploid asexual females of *C. gibelio* were selected on the basis of the following criteria: Basemean > 10, and a padj value < 0.05. For KEGG pathway analysis, no filtering based on log2 fold change was applied. Gene functions were investigated using the biological databases Uniprot [69], KEGG [61], Zfin [70] and GeneCards [71]. Principal component analysis (PCA) was performed using the DESeq2 R package [59]. For PCA based on reproduction-associated genes, a set of 208 reproduction genes was selected using the BioMart tool of Ensembl [65].

### Gene selection and real-time quantitative PCR

Based on the results of an NGS approach and published studies [20, 66–68], as well as the presence of appropriate GO and KEGG terms, candidate reproduction-associated genes were selected for the further analyses of gene expression. A-tubulin (*A-tub*) was used as a housekeeping gene to normalize variation in the gene expression, this gene was previously reported to be stable in fish ovary [72]. The Reference Gene Selection Tool from Bio-Rad CFX Maestro software (Bio-Rad), based on geNorm software principles [73] with an algorithm to normalize the Cq of each gene against the Cq values of the reference gene, was used. A total of 20 biologically-relevant genes were selected from transcriptomic outputs using published studies, and the expressions of 17 of them were validated by real-time quantitative PCR (qPCR). Three genes were excluded because of the amplification of unspecific products. Primers were designed using Primer Blast [74] at the exon-exon junction. A summary of the genes analysed, and their primer sequences are presented in Table 1.

**Table 1:** List of the target genes selected from RNA seq and the housekeeping gene analysed using RT-qPCR, and their respective primer sequences

Gene name	Gene description	Forward/reverse primers (5’->3’)	Amplicon size	
*A-TUB*	Alpha-Tubulin	TGCCAACTACGCCCG AGAGGTGAAACCAGAGCC		
*PIWIL2*	Piwi-Like Protein 2	TGACACCAACGGTTGCCA	81	
		CCCCCGTCCAAGAGGT		
*ZPE3L2*	Zona Pellucida Sperm-Binding Protein 3-Like	TTCTTTGCCAATGGGTGGCT	92	
		TCCCACTGAAAACACCTTCCT		
*RASA1B*	Ras Gtpase-Activating Protein 1	GGTTGTGGGTGACGAATGTC	97	
		CCATGAAACCAGGCTTTCCC		
*HRASAL*	Gtpase Hras	TCCGGGGAATCAGAGGTTGA	136	
		GGGGTCGTATTCGTCCACAA		
*ZP3EL1*	Zona Pellucida Sperm-Binding Protein 3-Like	TCTCTGCTAATGGTTGGGTGT	129	
		CTGGTCACTTCCTCTTCGGT		
*SPO11*	SPO11, Initiator of Meiotic Double Stranded Breaks	AGTACGGCTCACGGTCTCTG	117	
		TAAGCGTTTCCTCTGGGACTC		
*SYCE1*	Synaptonemal Complex Central Element Protein 1	CCCTACAGTTGGAGGGTACA	107	
		GTTCTGCTCAAGCTGCCTTTG		
*C1ORF146*	Chromosome 31 C1orf146 Homolog	CAAGCCCCAGTCTACGGAAA	141	
		GGTTTACTTGTGGCCTTCGC		
*SPINBZL*	Spindlin-Z-Like	AAGAGCTCTCACAAGCACAAA	136	
		CTTGGACTAGTACGGTCCCC		
*CAMSAP2A*	Calmodulin-Regulated Spectrin-Associated Protein 2	CCCAGACACCCGAAAAACAC	137	
		TCTTCTGGAACACTGTCTGTACC		
*DMRT2A*	Doublesex- And Mab-3-Related Transcription Factor 2	AGCAAGCGACAGAGGACAAA	91	
		GTTGATGGACGAATGTGCCG		
*NCOA2L*	Nuclear Receptor Coactivator 2	TTGCTGCTGAGTAATAACGACTG	141	
		TTTCCCCGACAGCACTCATC		
*RNF212*	Ring Finger Protein 212	CTTCGTGTCTCCTGGTCCTG	115	
		CAGACACCCTGTTTTCCTCTCT		
*SOX8L*	Transcription Factor SOX-8	CAACAGCTCCACGGTGCTCA	112	
		TGGTGTTATCCGATGCACGC		
*ALDH1A3*	Aldehyde Dehydrogenase 1 A3	GAAAACCATGCCAGTCGATGA	141	
		GTGTTCCCGCAGGCCAAA		
*CALM3A*	Calmodulin 3a	TAGACACGTTTATCGCACGGG	83	
		AACGCCTCCTTGAACTCAGC		
*BUC*	Bucky Ball	GGACCTCAGGATCAAGGGAG	106	
		CTTCGTGGCCTTTGTTGGTG		

Reverse transcription following total RNA extraction from preserved samples of gonadal tissues stored in RNAlater was performed using High-Capacity RNA-to-cDNA Kit (Applied Biosystems by Thermo Fisher Scientific) according to the manufacturer’s instructions. The suitability of primers, their optimal annealing temperatures and amplicon lengths, and the specificity of the amplification of all selected genes were verified by classical PCR for representative samples of all fish groups. The PCR reaction mix (10 ul) contained 5 µl of prepared cDNA, 1 x Taq Buffer with (NH4)_2_SO_4_, 1.5 mM MgCl_2_, 200 µM of each dNTP, 0.4 µM of forward and reverse primers (Table 1), 1 U of Taq DNA polymerase (Thermo Fisher Scientific), and nuclease-free water. PCR was run under the following conditions: initial denaturation at 95˚C for 4 min; 30 cycles of 95˚C for 30 s, an optimization gradient of 40–65˚C for 30 s, 72˚C for 45 s; and a final amplification at 72˚C for 10 min. At least 5 samples from each fish group were used for the test. Three replicates for each sample were included in the qPCR analysis.

Real-time qPCR was performed using the LightCycler 480 II Real-Time PCR System (Roche Diagnostics) and LightCycler 480 SYBR Green I Master chemistry (Roche). The reaction mixture (final volume 20 µl) consisted of 10 µl of 2x SYBR Green I Master, 1 µl of each primer, 3 µl of dd H_2_O, and 5 µl of cDNA template. To test the reaction efficiency and to obtain the standard amplification curve, templates were prepared by means of six serial decimal dilutions of the cDNA of representatives of each fish group. Reactions were run on a LightCycler 480 Instrument II under the following conditions: 95˚C for 5 min; 45 cycles of 95˚C for 10 s, 55˚C for 10 s, and 72˚C for 10 s; melt curve 55˚C → 95˚C (increment 0.5˚C)/5 s. In each run plate, together with samples run in triplicates, one negative control, in which RNase/DNase-free water was used instead of the cDNA and *A-tub* as the reference gene, was analysed. LightCycler 480 software 1.5.1 was used for analyses of qPCR outputs. The relative expression value of the differentially expressed target gene – the normalized expression – was computed using the ΔΔCq method. Differences in gene expression between sexual and asexual females were statistically evaluated. The sequences of the primers used in this analysis are listed in Table 1.

## Results

### Next generation sequencing and assembly and SNPs analysis of ***C. gibelio***

**Fig. 1:**
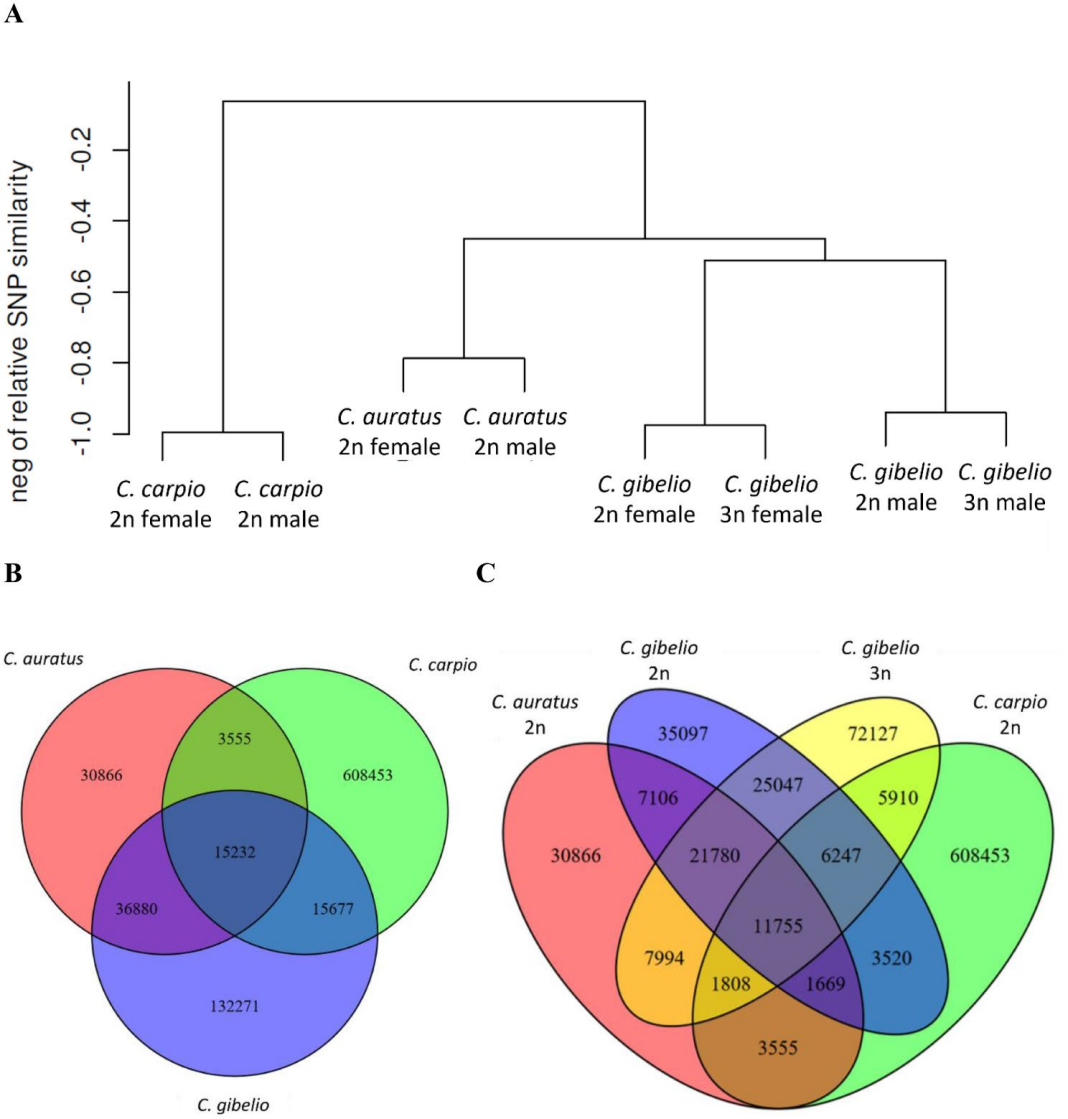
(**A**) Dendrogram of the hierarchical clustering of different lineages based on the degree of SNP similarity. Venn diagrams of the numbers of SNPs shared (**B**) between *C. gibelio*, *C. auratus* and *C. carpio*, and (**C**) between *C. auratus*, *C. carpio*, and diploid and triploid females of *C. gibelio*

The sequencing of four to five diploid males and females from *C. gibelio*, *C. auratus* and *C. carpio*, and triploid females and males of *C. gibelio* yielded from 8 M to 17 M raw reads per individual (**Additional file 1**). The number of mapped reads varied between 5 M and 12 M. Across individual samples, from 51 to 83% of reads were uniquely mapped, and from 12% to 22% of reads were multimapped. A total of 857,874 SNPs were identified in the transcriptomes of the eight fish groups (males and females of the three species including both triploid and diploid forms of gibel carp). Clustering analysis based on SNP numbers showed that *C. gibelio* and *C. auratus* are closely related and that asexual *C. gibelio* and sexual *C. gibelio* are conspecific (Fig. 1A). Specifically, the proportion of SNPs shared between *C. gibelio* and *C. auratus* was 2.35 times higher than the proportion of SNPs shared by *C. gibelio* and *C. carpio* (Fig. 1B). However, *C. carpio* and *C. auratus* shared only 3555 SNPs. The sexual diploid and asexual triploid individuals of *C. gibelio* were more similar to each other than to *C. auratus* or *C. carpio* and both forms shared a similar number of SNPs with *C. auratus* (Fig. 1C).

### Differential gene expression analysis

**Fig. 2:**
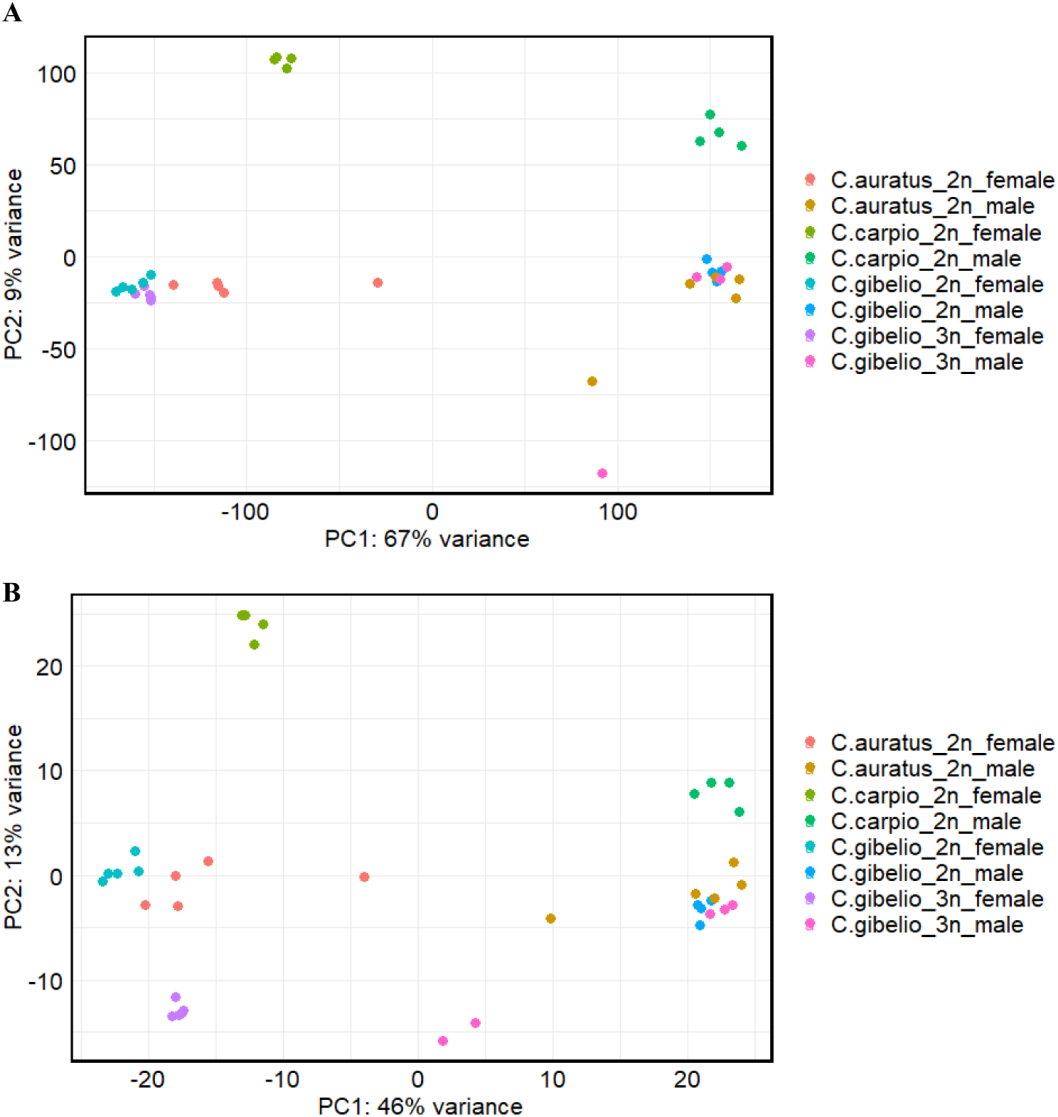
Principal component analysis (PCA) of normalized RNAseq read counts between the diploid males and females of *C. gibelio*, *C. auratus* and *C. carpio* and the triploid females and males of *C. gibelio* for all genes (**A**) and a set of 208 randomly selected reproductive genes (**B**), on the first two principal components

The transcriptome profiles of the females and males of *C. gibelio*, *C. auratus* and *C. carpio* were analysed (Fig. 2). Both reproductive forms – asexual and sexual – were included for *C. gibelio*. In all cases, the biological replicates of same sex, ploidy level, and species tend to be more similar to each other. PCA based on transcriptome-wide gene expression (Fig. 2A) showed differences in transcriptome profiles between sexes of the same species, these separated by PC1, and a similarity between the transcriptome profiles of the asexual females of *C. gibelio* and the sexual females of *C. gibelio* and *C. auratus*. However, even the females of *C. auratus* were separated from *C. gibelio* by PC1. Likewise, the transcriptomes of the diploid and triploid males of *C. gibelio* and *C. auratus* also tended to be similar to each other. According to the transcriptome profiles, the males and females of *C. carpio* were separated from the other fish groups by PC2. To compare the expression levels of reproduction-related genes among fish groups, a total of 208 genes related to reproduction were selected. This set of reproductive genes led to a similar grouping of species and sexes, as revealed by all of the transcriptomic data; however, the asexual triploid females *C. gibelio* were more separated from the sexual ones of by PC2 (Fig. 2B).

The numbers of non-differentially and differentially expressed genes are shown in Table 2. For all comparisons, the number of upregulated genes in *C. gibelio* asexual females was higher than the number of downregulated genes or similar to the number of downregulated genes. Comparison of the asexual and sexual females of *C. gibelio* revealed 1728 differentially expressed genes (DEGs). The numbers of upregulated and downregulated genes are shown in Table 2. The number of DEGs in asexual *C. gibelio* females was lower compared to sexual females in every species than compared to males of the same species. The number of DEGs between asexual *C. gibelio* females and *C. auratus* females and males and the number of DEGs between asexual *C. gibelio* females and *C. carpio* females and males was higher when compared to the number of DEGs between asexual *C. gibelio* females and sexual *C. gibelio* females and males (Table 2).

**Table 2:** Number of non-differentially expressed genes and differentially expressed genes (down- and upregulated) in the triploid asexual females of *C. gibelio* compared to each of the diploid sexual males and females of *C. gibelio*, *C. auratus* and *C. carpio*

	C. gibelio 2n females	C. gibelio 2n males	C. gibelio 3n males	C. auratus 2n females	C. auratus 2n males	C. carpio 2n females	C. carpio 2n males
Non-differentially expressed gens	46,058	43,659	22,363	47,174	46,089	42,435	42,751
Downregulated genes in asexual females	782	3836	13,603	4214	8298	6773	9185
Upregulated genes in asexual females	946	10,944	7634	4704	11,841	6733	10,818

### GO enrichment analysis

**Fig. 3:**
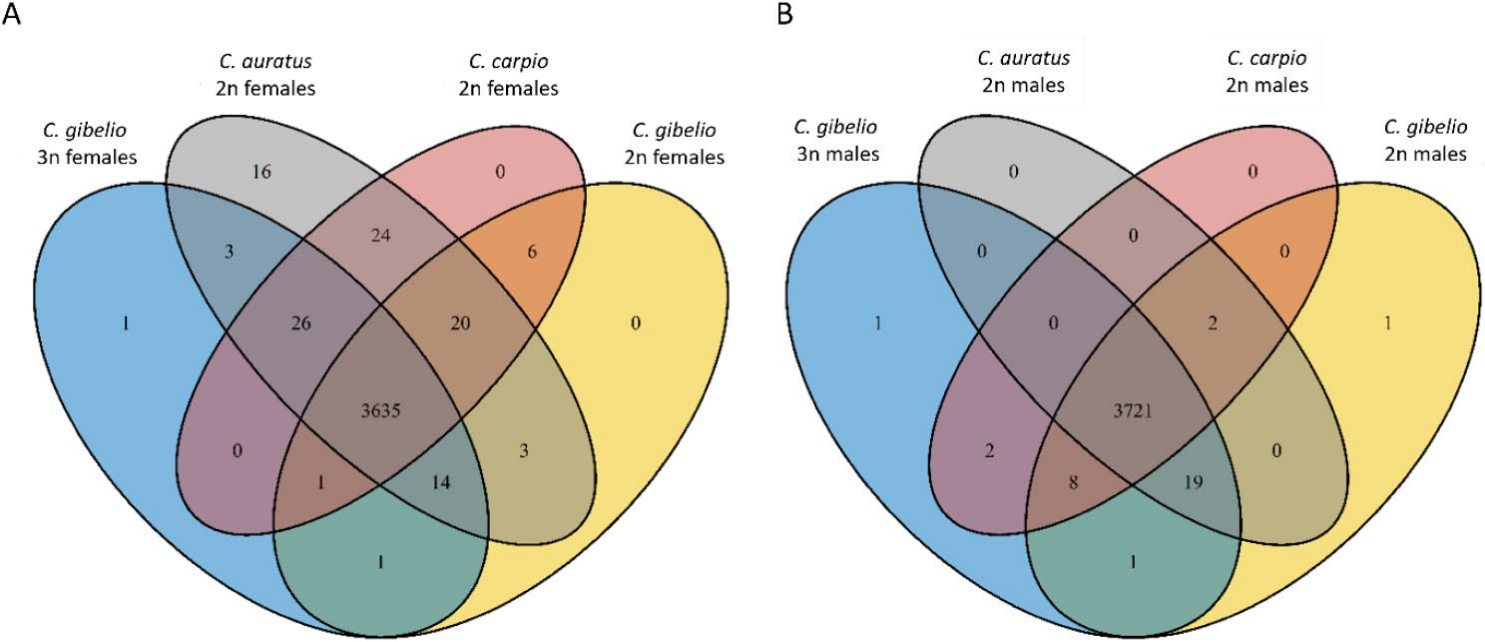
Venn diagram of Gene Ontology terms for the females (**A**) and males (**B**) of *C. gibelio*, *C. auratus* and *C. carpio*, including the triploid asexual females of *C. gibelio* and the triploid males of *C. gibelio*. Total numbers of unique and shared identified GO terms are indicated

The full transcriptomes of the three species were functionally annotated to 3747 GO terms for females and 3755 GO terms for males. A total of 3635 were shared by all female lines, and 3721 were shared by all male lines. 30 GO terms identified in asexual females of *C. gibelio* were not identified in the sexual females of *C. gibelio*, and 30 GO terms identified in the sexual females were not present in the asexual females of *C. gibelio*. Furthermore, 3 GO terms were identified in diploid males of *C. gibelio* but not in triploid males, and 3 GO terms were identified in triploid males of *C. gibelio* but not in diploid males (Fig. 3).

**Fig. 4:**
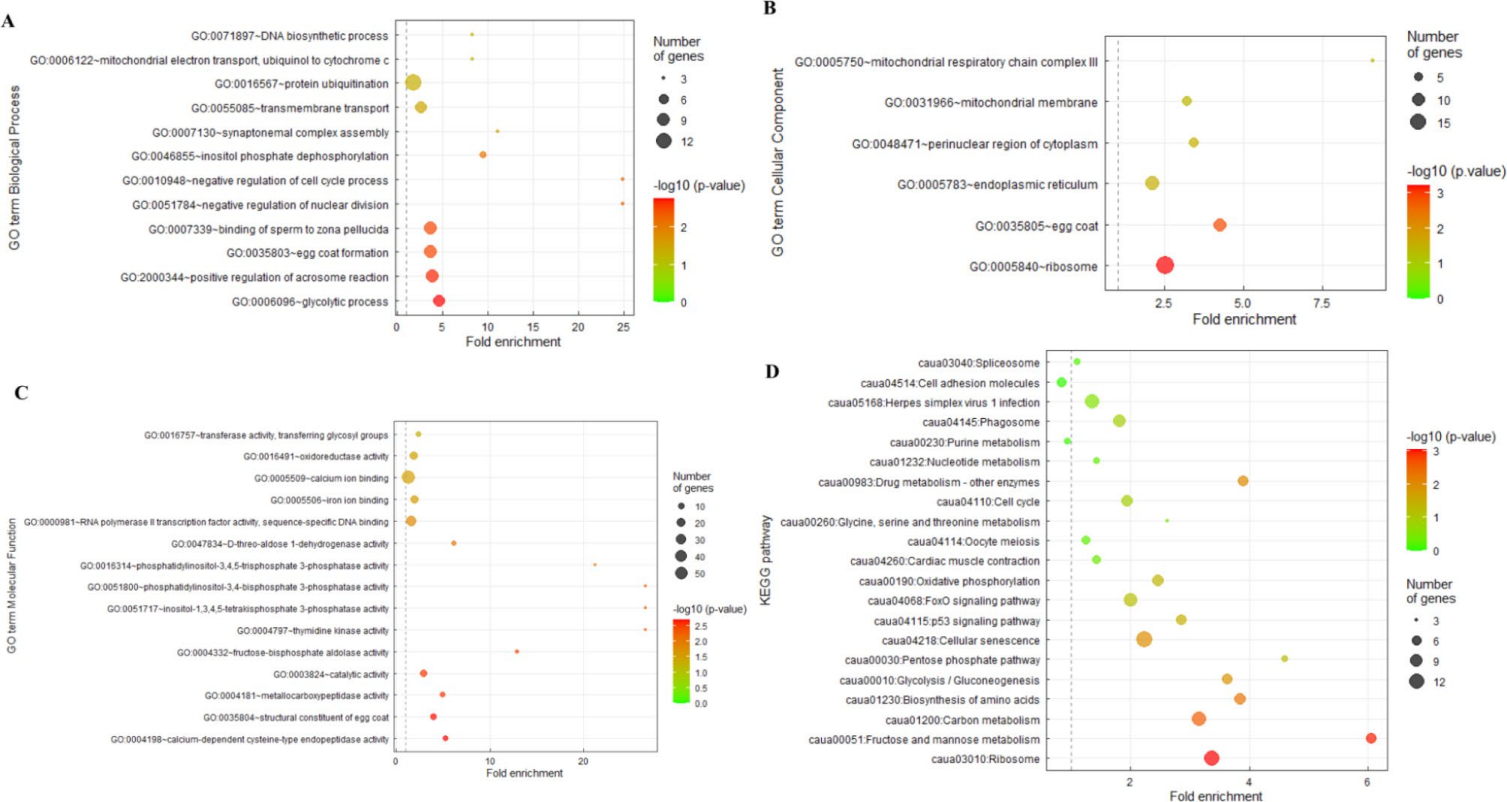
Dot plot of GO terms enrichment analysis in the biological process (**A**), cellular component (**B**), molecular function (**C**), and KEGG pathway (**D**) categories. The x-axis represents the fold enrichment (the number of DEGs in the GO term / the number of all DEGs)/(the number of genes annotated in this pathway/ the number of the genes annotated in all pathways). The y-axis corresponds to the enriched GO terms. The magnitude of dots represents the number of DEGs in the GO term, and the color corresponds to the -log10 of the p-value

Transcriptomes of sexual and asexual females were compared and investigated for pathway enrichment using overrepresentation analysis. Of the total of 1728 DEGs, 1471 were successfully annotated to the Gene Ontology (GO) and KEGG databases. A total of 809 were upregulated in asexual females in comparison to sexual females, and 662 downregulated. The significantly enriched GO terms are presented in Fig. 4. In the biological process category, we identified GO terms associated with gametogenesis and cell cycle control, including egg coat formation (GO:0035803), the binding of sperm to zona pellucida (GO:0007339), the positive regulation of acrosome reaction (GO:2,000,344), synaptonemal complex assembly (GO:0007130), the negative regulation of nuclear division (GO:0051784), and the negative regulation of cell cycle process (GO:0010948). In the cellular component category, the most enriched terms included egg coat (GO:0035805). In the molecular function category, they included the structural constituent of egg coat (GO:0035804) and calcium ion binding (GO:0005509). The significantly enriched KEGG pathways included oocyte meiosis (caua04114), and cell cycle (caua04110).

### Meiosis-associated genes

To determine whether meiotic pathways are disrupted in asexual females of *C. gibelio*, we first analysed the differences in expression levels of the meiosis-associated genes between sexual and asexual females following refs [66–68, 75, 76]. Of the set of 40 meiosis-associated genes, almost all were detected in both asexual and sexual females; however, *pms1* was not detected in most sexual and asexual females, and *hormad2* was not detected in any sexual or asexual individual. Hence, the meiotic pathways did not appear to be disrupted in asexual females. Seven genes were significantly differently regulated. *Spo11, msh2*, *pds5b* and *stag1a* displayed higher expression levels in sexual females when compared to asexual females, as well as *rec114*, which was close to significance (padj = 0.07). In contrast, *rad1*, one *rad51b* homologue and *slc39a1* were significantly more expressed in asexual females. The other meiosis-associated genes, including meiotic nuclear division 1 (*mnd1), dmc1*, the double strand break repair *rad1* and several *rad51* homologues, did not show significant gene expression differences (Table 3).

**Table 3:** List of meiosis-associated genes with their expression levels in sexual and asexual females of *C. gibelio*

Ensembl ID	Gene name	Gene description	L2fc	padj
ENSCARG00000016377*	*spo11*	SPO11 initiator of meiotic double stranded breaks	-1.75	5.36e-6
ENSCARG00000024429	*hormad1*	HORMA domain containing 1	0.30	0.22
ENSCARG00000034047	*mnd1*	Meiotic nuclear division 1	-0.4	0.24
ENSCARG00000050983	*mlh1*	MutL homolog 1	-0.6	0.61
ENSCARG00000069004	*mlh3*	MutL homolog 3	-0.1	0.87
ENSCARG00000021963	*pms1*	PMS homolog 1	-0.33	NA
ENSCARG00000010121	*pms2*	PMS homolog 2	0.02	0.97
ENSCARG00000007723	*dmc1*	DNA meiotic recombinase 1	-0.38	0.55
ENSCARG00000038661*	*msh2*	MutS homolog 2	-0.85	5.83e-6
ENSCARG00000047192	*msh4*	MutS homolog 4	0.79	0.52
ENSCARG00000015097	*msh5*	MutS homolog 5	-0.01	0.99
ENSCARG00000011896	*msh6*	MutS homolog 6	-0.36	0.18
ENSCARG00000011987*	*rad1*	Rad1 cohesin complex component	1.43	8.11e-6
ENSCARG00000026371	*rad21*	Rad21 cohesin complex component	0.75	0.37
ENSCARG00000022693	*rad50*	Rad50 double strand repair protein	-0.25	0.70
ENSCARG00000002053	*rad51c*	Rad51 recombinase	0.30	0.85
ENSCARG00000010638*	*rad51b*	Rad51 recombinase	0.81	0.03
ENSCARG00000018365	*rad51d*	RAD51 recombinase	0.29	0.35
ENSCARG00000027817	*rad51*	RAD51 recombinase	-0.71	0.55
ENSCARG00000056842	*rad51*	RAD51 recombinase	0.69	0.63
ENSCARG00000064885	*rad51b*	RAD51 recombinase	0.31	0.69
ENSCARG00000047813	*rad51*	RAD51 recombinase	0.14	0.92
ENSCARG00000004144	*rad51*	RAD51 recombinase	-0.02	0.96
ENSCARG00000045888	*rad52*	Rad52 DNA repair protein	0.01	0.96
ENSCARG00000039864	*rec8*	Rec8 meiotic recombination protein	1.27	0.59
ENSCARG00000032822	*rec114*	Rec114 meiotic recombination protein	-1.4	0.07
ENSCARG00000057676	*smc1b*	Structural maintenance of chromosome 1b	1.02	0.13
ENSCARG00000039472	*smc1a*	Structural maintenance of chromosome 1a	-0.11	0.74
ENSCARG00000005430	*smc2*	Structural maintenance of chromosome 2	-0.24	0.4
ENSCARG00000055515	*smc3*	Structural maintenance of chromosome 3	-0.21	0.51
ENSCARG00000010356	*smc4*	Structural maintenance of chromosome 4	0.13	0.90
ENSCARG00000042929	*smc5*	Structural maintenance of chromosome 5	-0.13	0.75
ENSCARG00000021147	*pds5a*	PDS5 cohesin associated factor B	-0.17	0.75
ENSCARG00000017951*	*pds5b*	PDS5 cohesin associated factor B	-0.98	4.7e-6
ENSCARG00000001906*	*stag1a*	Cohesin subunit SA 1 A	-0.96	0.02
ENSCARG00000022475	*stag1b*	Cohesin subunit SA 1B	-0.24	0.47
ENSCARG00000052961	*mre11*	Double strand break repair nuclease	-0.04	0.92
ENSCARG00000018605	*hfm1 (mer3)*	Helicase for meiosis 1	-0.27	0.81
ENSCARG00000053878*	*slc39a1*	Solute carrier family 39A1	0.65	0.04
ENSCARG00000062463	*mus81*	Crossover junction endonuclease MUS81	0.10	0.77

A positive log2 fold change (l2fc) indicates transcripts that were more abundant in asexual females when compared to sexual females. A negative log2 fold change indicates transcripts that were more abundant in sexual females when compared to asexual females. Asterisks indicate significant difference in expression levels between sexual and asexual females (padj < 0.05)

### Identification of differentially expressed genes in sexual and asexual females of***C. gibelio***

**Fig. 5:**
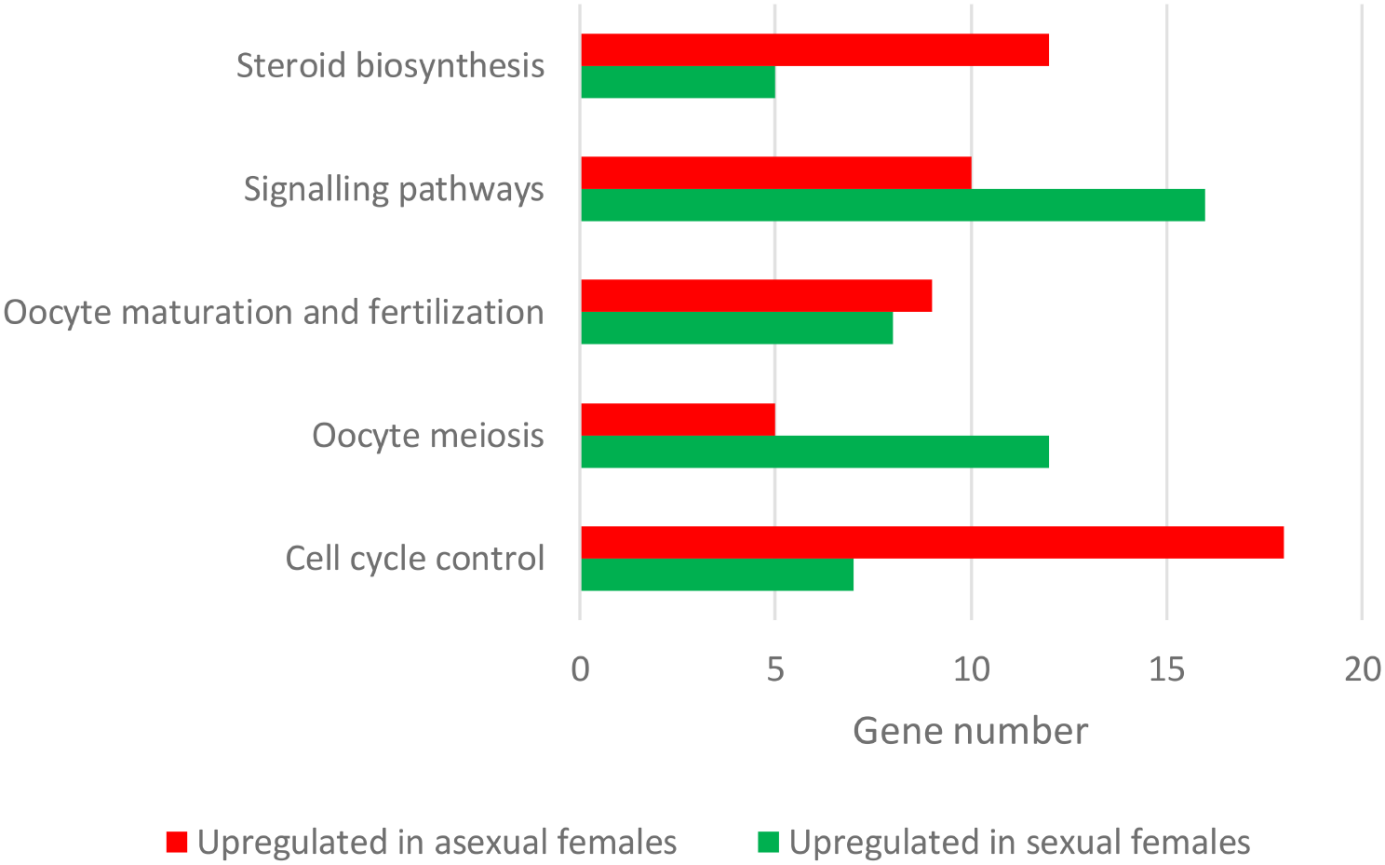
Summary of the number of genes upregulated in asexual females or in sexual females of *C. gibelio* in reproduction-associated pathways

Among the 1728 differentially expressed genes revealed by transcriptome profile analysis, we specifically focussed on the genes related to reproduction pathways revealed by GO and KEGG enrichment analyses and published studies [20, 66, 68]. We identified genes that were involved in reproduction pathways including cell cycle control, oocyte meiosis and maturation, and signalling pathways related to reproduction and sex differentiation (Fig. 5, see Table 4 for the list of the genes and their biological function).

**Table 4:** List of selected differently-expressed genes potentially involved in the reproduction of *C. gibelio*, including the description of gene function according to the biological databases Uniprot, KEGG, Zfin and GeneCards unless other references are mentioned

Ensembl ID	Gene name	Gene description	Gene function	l2fc	padj
ENSCARG00000024627	*acvr2ba*	Activin receptor 2B	Transduces activin signal from cell surface to cytoplasm	-1.73	***
ENSCARG00000025713	*akt1*	RAC-alpha serine/threonine-protein kinase	Meiotic maturation [137]	2.06	***
ENSCARG00000012651	*bambia*	BMP and activin membrane bound inhibitor receptor 2	TGF-β signal transduction	-2.18	***
ENSCARG00000010645	*bcl2*	Apoptosis regulator Bcl-2-like	Apoptosis regulation and oocyte development	1.73	*
ENSCARG00000036539	*bmp2b*	Bone morphogenetic protein 2-like	Growth factor involved in diverse cell processes including oocyte maturation	1.41	*
ENSCARG00000042808	*bmp8a*	Bone morphogenetic protein 8 A-like	Growth factor involved in diverse cell processes including oocyte maturation	-7.80	***
ENSCARG00000045704	*buc*	Bucky ball	Formation of the Balbiani body in the oocyte, establishment of oocyte polarity	-2.84	***
ENSCARG00000067925	*c1orf146*	Chromosome 31 c1orf146 homolog	Synaptonemal complex assembly and meiotic recombination	1.15	*
ENSCARG00000061657	*calm3a*	Calmodulin 3a	Fertilization Ca^2+^-dependant signal transduction pathway	-1.07	***
ENSCARG00000004753	*camk1gb*	Calcium/calmodulin-dependent protein kinase	Ca^2+^-dependant signal transduction pathway	1.42	*
ENSCARG00000025177	*camsap2a*	Calmodulin-regulated spectrin-associated protein 2	Sperm binding protein in males	3.68	***
ENSCARG00000044731	*ccna2*	Cyclin A2	Cell cycle control	2.44	***
ENSCARG00000066013	*ccnb2*	Cyclin-B2	Cell cycle control	1.02	**
ENSCARG00000026715	*ccnd2a*	Cyclin D2a	Cell cycle control	1.50	**
ENSCARG00000060407	*ccnf*	Cyclin F	Cell cycle control	4.68	***
ENSCARG00000058284	*cdk14*	Cyclin dependant kinase 14	Cell cycle control	1.54	***
ENSCARG00000063775	*cdk5rap1*	CDK5 regulatory subunit associated protein 1	Cell cycle control	1.12	***
ENSCARG00000030409	*clec*	C-type lectin	Cell surface receptor involved in cell communication during egg fertilization	-2.73	***
ENSCARG00000046375	*clk4*	Dual specific protein kinase CLK4	Sex differentiation	1.05	*
ENSCARG00000056466	*cpeb1a*	Cytoplasmic polyadenylation element binding	Cell proliferation regulation	-5.69	***
ENSCARG00000018125	*cxcl12a*	Chemokine ligand 12a	Development of oocytes	-3.40	**
ENSCARG00000069389	*cyp19a1a*	Cytochrome P450 19 A 1a	Ovarian follicle development and female sex determination	1.81	**
ENSCARG00000036303	*ddx20*	DExD-box helicase 20	Ovarian development and function [138]	-1.06	***
ENSCARG00000010183	*ddx52*	DExD-box helicase 52	Cell cycle control	-1.77	***
ENSCARG00000031374	*dmrt2a*	Doublesex and mab3 related transcription factor 2a	Female germ cell development and oogenesis [139]	2.22	***
ENSCARG00000062724	*dmrta2*	Doublesex and mab3-related transcription factor 2	Female germ cell development and oogenesis	2.02	***
ENSCARG00000008338	*e2f1*	E2F transcription factor 1	Cell cycle control	3.11	***
ENSCARG00000037330	*fbxo15*	F-box protein 15	Embryonic development	3.60	***
ENSCARG00000050933	*fbxo28*	F-box only protein 28-like	Cell cycle control and substrates degradation in meiosis [140]	1.03	*
ENSCARG00000056526	*emi1 (fbxo5)*	F-box protein	Regulation of the APC in mitosis and meiosis	-7.00	***
ENSCARG00000013439	*emi1 (fbxo5)*	F-box protein	Regulation of the APC in mitosis and meiosis	-8.26	***
ENSCARG00000018093	*emi1 (fbxo5)*	F-box protein	Regulation of the APC in mitosis and meiosis	-7.47	***
ENSCARG00000028524	*fgf18a*	Fibroblast growth factor 18	Oocyte nuclear maturation [141]	4.97	***
ENSCARG00000016389	*fgf4*	Fibroblast growth factor 4	Oocyte differentiation [142]	3.03	***
ENSCARG00000009251	*fmnl2a*	Formin-like 2 A	Cell division and polarity	4.13	***
ENSCARG00000056928	*gadd45ba*	Growth arrest and DNA damage 45 ba	Cell cycle control [143]	1.40	**
ENSCARG00000027726	*grapb*	GRB2-related adapter protein B	Oocyte meiosis	2.69	*
ENSCARG00000027104	*Grb2*	Growth factor receptor bound protein 2	Signal transduction, GnRH signalling pathway	-0.59	**
ENSCARG00000027108	*h2af1o*	Histone 2 A F1o	Oocyte-specific histone H2A variant	2.07	**
ENSCARG00000033210	*hbegf*	Heparin binding EGF like growth factor	GnRH signalling pathway	-0.93	***
ENSCARG00000013938	*hrasa*	Gtpase hras-like	Cell division regulation in response to growth factors	-1.19	***
ENSCARG00000021215	*hsd17b1*	Hydroxysteroid 17-beta dehydrogenase 1	Estrogen activation and androgen inactivation	1.23	*
ENSCARG00000005210	*inha*	Inhibin Subunit Alpha	Ovarian development [144]	1.32	**
ENSCARG00000015712	*lbh*	LBH regulator of WNT signalling pathway	Oocyte maturation in Gibel carp	-1.58	***
ENSCARG00000017091	*lhcgr*	Luteinizing hormone/choriogonadotropin receptor	Gonad development and differentiation	-1.64	*
ENSCARG00000056775	*mad2l2*	Mitotic arrest deficient 2 like 2	Spindle assembly checkpoint protein	2.52	***
ENSCARG00000062672	*mad2l2*	Mitotic arrest deficient 2 like 2	Spindle assembly checkpoint protein	2.36	**
ENSCARG00000025045	*mapk8ip3*	MAPK 8 interacting protein 3	Involved in FSH signalling pathway	1.67	**
ENSCARG00000019928	*mcm5*	Minichromosome maintenance complex component 5	Cell cycle regulation	-1.44	**
ENSCARG00000048754	*mcm9*	Minichromosome maintenance complex component 9	Repair of double stranded DNA breaks	-1.22	***
ENSCARG00000035099	*ncoa2*	Nuclear receptor coactivator 2-like	Activation of steroid receptors	-1.67	***
ENSCARG00000039143	*nqo1*	NAD(P)H quinone dehydrogenase 1	Cell cycle control [145]	-1.29	*
ENSCARG00000020971	*oxtr*	Oxytocin receptor	Control of reproductive systems	1.72	**
ENSCARG00000004805	*piwil2*	Piwi-like protein 2	Meiotic differentiation of spermatocytes	-1.50	***
ENSCARG00000061907	*pkcdb*	Protein kinase C DB	Component of the GnRH signalling pathway [146]	1.44	**
ENSCARG00000028187	*pkcba*	Protein kinase C BA	Component of the GnRH signalling pathway [146]	2.68	*
ENSCARG00000013369	*plcb4*	Phospholipase C beta 4	Sperm cell fertilization [147]	-2.62	***
ENSCARG00000034226	*plcd4b*	Phospholipase C delta 4b	Sperm cell fertilization	-1.99	***
ENSCARG00000049505	*pld4*	Phospholipase D family member 4	GnRH signalling pathway	1.91	*
ENSCARG00000044904	*plxnb1a*	Plexin-B1-like	Follicular development [148]	1.73	**
ENSCARG00000011987	*rad1*	Rad1 cohesin complex component	Cell cycle checkpoint protein	1.43	***
ENSCARG00000036380	*rasa1a*	Ras GTPase-activating protein 1-like	Cell division regulation in response to growth factors	-1.20	*
ENSCARG00000013635	*rasa1b*	Ras GTPase-activating protein 1-like	Cell division regulation in response to growth factors	-1.49	***
ENSCARG00000053044	*rasl11b*	Ras-like protein family member 11B	Sexual reproduction	2.15	*
ENSCARG00000014802	*rassf7b*	Ras association domain-containing protein 7-like	Cell cycle control	-8.71	***
ENSCARG00000012505	*rbpms2b*	RNA-binding protein with multiple splicing 2-like	Ovarian development [149]	-1.09	***
ENSCARG00000019039	*rfc3*	Replication factor C3	Cell cycle progression [150]	0.97	*
ENSCARG00000045179	*rfc4*	Replication factor C4	Cell cycle progression	-1.03	*
ENSCARG00000022178	*rnf212*	Ring finger protein 212	Meiotic recombination	5.34	***
ENSCARG00000006237	*sbk3*	Serine/threonine-protein kinase	Female meiosis chromosome segregation	-7.64	***
ENSCARG00000044509	*setd7*	SET domain containing 7	Sex differentiation	-2.09	***
ENSCARG00000018258	*smad2*	Mothers against decapentaplegic homolog 2	TGF-β signalling pathway	-1.18	***
ENSCARG00000064397	*smad6a*	Mothers against decapentaplegic homolog 6-like	TGF-β signalling pathway	-1.60	**
ENSCARG00000058624	*sox8a*	SRY-box transcription factor 8a	Male sex determination	3.88	***
ENSCARG00000007149	*spag1a*	Sperm-associated antigen 1 A-like	Sperm cell fertilization	-2.39	***
ENSCARG00000000918	*spinb*	Spindlin-Z-like	Gametogenesis	1.36	***
ENSCARG00000017015	*spinw*	Spindlin-W-like	Gametogenesis	-1.28	**
ENSCARG00000016377	*spo11*	SPO11 initiator of meiotic double stranded breaks	Meiotic recombination	-1.75	***
ENSCARG00000001906	*stag1a*	Cohesin subunit STAG1A	Sister chromatid cohesion complex	-0.97	**
ENSCARG00000007335	*stk32c*	Serine/threonine-protein kinase 32 C	Regulation of meiosis	-1.39	***
ENSCARG00000018451	*syce1*	Synaptonemal complex element 1	Part of the synaptonemal complex	-1.56	***
ENSCARG00000041319	*tgfb1a*	Transforming growth factor beta-1-like	Diverse pathways including gonadal growth	5.97	***
ENSCARG00000003682	*tgfb1a*	Transforming growth factor beta-1-like	Diverse pathways including gonadal growth	5.78	***
ENSCARG00000049821	*uhrf1*	Ubiquitin-like containing PHD and RING finger domain 1	Cell cycle control, epigenetic regulation	2.32	***
ENSCARG00000031722	*wnt5b*	Wnt-5B	Ovarian development	2.72	***
ENSCARG00000042293	*wnt7bb*	Protein Wnt-7b-like	Ovarian development	3.38	***
ENSCARG00000029293	*zp3el*	Zona Pellucida Sperm-Binding Protein 3-Like	Sperm binding glycoprotein of the egg coat	1.76	***
ENSCARG00000015906	*zp3el*	Zona Pellucida Sperm-Binding Protein 3-Like	Sperm binding glycoprotein of the egg coat	1.39	***
ENSCARG00000007183	*zpel3*	Zona Pellucida Sperm-Binding Protein 3-Like	Sperm binding glycoprotein of the egg coat	-1.08	**
ENSCARG00000042829	*zpel3*	Zona Pellucida Sperm-Binding Protein 3-Like	Sperm binding glycoprotein of the egg coat	-1.76	***
ENSCARG00000054343	*sgo*	Shugoshin 1	Chromosome cohesion during cell division	-0.68	***
ENSCARG00000000832	*plkk1*	Serine/threonine-protein kinase 10-like	Cell cycle control and meiosis regulation	-1.69	**
ENSCARG00000055958	*ccnd3*	Cyclin D3	Cell cycle control	-1.03	**
ENSCARG00000038200	*fmnl1a*	Formin-like 1 A	Cell division and polarity	2.27	***
ENSCARG00000032499	*aurka (= eg2)*	Aurora kinase A	Cell cycle control, spindle assembly during chromosome segregation	0.91	**
ENSCARG00000058511	*pp1*	Ser/thr-protein phosphatase PP1 catalytic subunit	Oocyte meiosis	-0.66	***
ENSCARG00000005591	*cdc20*	Cell division cycle protein 20	Cell cycle and meiosis regulation	0.69	*
ENSCARG00000030662	*cdc25*	Cell division cycle protein 25	Cell cycle and meiosis regulation	-0.65	**
ENSCARG00000064615	*fzr1b*	Fizzy and cell division cycle 20 related 1	Cell cycle and meiosis regulation	-0.56	*
ENSCARG00000002809	*creb (= atf4b)*	cAMP-dependent transcription factor ATF-4	GnRH signaling pathway	-0.82	***
ENSCARG00000022063	*acvr1*	Activin receptor 1	TGF-B signaling pathway	-0.74	*
ENSCARG00000045643	*fk*	Delta14-sterol reductase	Steroid biosynthesis	1.89	*
ENSCARG00000066569	*ste1 (= sc5d)*	Lathosterol oxidase-like	Steroid biosynthesis	1.21	**
ENSCARG00000063352	*erg3*	Sterol desaturase	Steroid biosynthesis	1.22	**
ENSCARG00000040844	*hyd1*	Cholestenol Delta-isomerase	Steroid biosynthesis	-1.21	*
ENSCARG00000069123	*cyp27b1*	Cytochrome P450 27 b 1	Steroid biosynthesis	-2.09	***
ENSCARG00000018413	*hsd3b*	Beta-hydroxy-Delta5-steroid dehydrogenase	Steroid biosynthesis	3.03	***

A positive log2 fold change indicates transcripts that were more abundant in asexual females when compared to sexual females. A negative log2 fold change indicates transcripts that were more abundant in sexual females when compared to asexual females. Abbreviations: APC: anaphase promoting complex, BMP: bone morphogenic protein, CDK: cyclin dependant kinase, GnRH: gonadotropin releasing hormone, TGF: transforming growth factor. Asterisks indicate statistically significant differences between sexual and asexual females of *C. gibelio* based on padj value: *padj < 0.05, **padj < 0.01, ***padj < 0.001

Asexual females retained detectable expressions of all the reproduction-associated genes identified. However, several genes involved in cell cycle control were differently expressed between asexual and sexual females. Asexual females upregulated genes of the Cyclin family, such as *ccna2, ccnb2*, *ccnd2a* and *ccnf* as well as *cdk14*, a member of the cyclin dependant kinase family (Additional file 2). They also upregulated *growth arrest and DNA damage protein 45 alpha B* (*gadd45ab*), the activator *e2f1*, *mitotic arrest deficient 2 like 2* (*mad2l2*), *ring finger 212* (*rnf212*), *aurora kinase* a (*aurora a), cell division cycle protein 20 (cdc20)*, the apoptosis regulator *bcl2*, *serine threonine kinase 1* (*akt1)*, and *cdk5rap1*, which encodes the CDK5 regulatory-subunit-associated protein 1 (see Table 4 for their functions). Members of the formin family, *fmnl1a* and *fmnl2a*, were also upregulated in asexual females, as well as the spindlin *spinb*, while *spinw* was downregulated. Sexual females also upregulated two genes encoding ATP-dependant RNA helicases, *ddx20* and *ddx52*; as well as *nqo1*, which encodes the NAD(P)H quinone dehydrogenase 1; *rassf7b*, which encodes the ras-associated domain-containing protein 7b; and *stag1a*, which encodes a cohesin subunit.

Sexual females upregulated genes involved in oocyte meiosis such as *shugoshin 1 (sgo1)*; *serine/threonine kinase 10* (*plkk1*); *phosphatase 1 (pp1*); *serine/threonine-protein kinase 32 C* (*stk32c*); *cytoplasmic polyadenylation element binging* (*cpeb*); *syce1*, which encodes a protein of the synaptonemal complex that forms between homologous chromosomes during meiosis; and several gene copies of *early mitotic inhibitor 1 (emi1*, also known as *fbxo5)* (Additional file 3, Table 4). They also upregulated genes involved in DNA mismatch repair, including *rfc4 (replication factor C subunit 4*) and genes that encode components of the minichromosome maintenance protein complex, *mcm5* and *mcm9*. Inversely, asexual females upregulated *c1orf146*, involved in synaptonemal complex assembly.

Concerning oocyte maturation pathways (Additional file 4), sexual females upregulated *bucky ball* (*buc*), *cell division cycle protein 25 (cdc25)*, *fizzy-related protein homolog 1b (fzr1b)*, *phospholipases cb4* and *cd4* (*plcb4* and *plcd4)*, and several gene copies of *zona-pellucida sperm-binding protein 3* (*zp3el*) (Table 4). On the other hand, asexual females upregulated *h2af1*, which encodes an oocyte-specific histone, and *uhrf1*, which encodes the oocyte specific cell cycle regulator E3 ubiquitin ligase. Members of the *fibroblast growth factor* (*fgf*) family were also upregulated. Several egg fertilization-related genes were differently regulated. *Calmodulin 3a* (*calm3a)*, *spag1a (sperm-associated antigen 1a-like*), and *clec*, which encodes a C-type lectin, were upregulated in sexual females (Additional file 3). *Camk1gb*, which encodes a calcium/calmodulin-dependent protein kinase, and *calmodulin-regulated spectrin-associated protein 2 (camsap2a)* were upregulated in asexual females (Table 4).

Genes involved in signalling pathways were also differentially regulated. Sexual females upregulated genes involved in the gonadotropin releasing hormone (GnRH) signalling pathway, which is important for female sexual differentiation (Additional file 5), such as *creb*, *heparin-binding egf-like growth factor (hbegf*), *growth factor receptor-bound protein 2 (grb2*), *rbpms2b*, involved in ovarian development, and members of the Ras/MAPK family, specifically, *hrasa, hrasb* and *rasa1b*, as well as *limb bud-heart (lbh)*, *bmp8* and *bambia* (Table 4). Asexual females upregulated *pkc*; *phospholipase d4b (pld4b); mapk8ip3*, involved in the FSH signalling pathway; the protein-kinase encoding gene *clk4*; *plexin b1a*; *bmp2b*; and members of the *wnt* family (*wnt5* and *wnt7*); as well as *fbxo15* and *fbxo28*, two members of the *fbxo* family (F-box with uncharacterized domains). Furthermore, components of the TGF-β (transforming growth factor) signalling pathway were differently regulated. *Tgf-β 1a* was upregulated in sexual females, while the activin receptors *acvr1* and *acvr2ba, bmp and activin membrane bound inhibitor activin receptor 2* (*bambia*), the receptor regulated *mothers against decapentaplegic homolog* (*smad2*) and the inhibitory *smad6* were downregulated (Additional file 6).

KEGG analysis identified DEGs involved in hormonal systems. Asexual females upregulated *cyp19a1a*, the *doublesex* and *mab3* related transcription factors *dmrta2* and *dmrt2a*, the sry-box transcription factor *sox8a*, *inhibin alpha* (*inha)*, and *oxtr*, encoding the oxytocin receptor **(**Table 4). Sexual females upregulated *piwil2*, *c-x-c motif chemokine 12* (*cxcl12)*, *nuclear receptor coactivator 2* (*ncoa2)*, and *luteinizing hormone/choriogonadotropin receptor (lhcgr)*. Several genes related to steroid biosynthesis were also found to be differently regulated between asexual and sexual females (Additional file 7). Asexual females upregulated *delta14-sterol reductase* (*fk)*; *lathosterol oxidase-like (ste1)*; *17beta-estradiol 17-dehydrogenase (hsd17b1*, 1.1.1.62); *β-hydroxy-δ5-steroid dehydrogenase* (*Hsd3b*); and genes encoding a glucuronosyltransferase (EC 2.4.1.17), a squalene synthase (EC 2.5.1.21), a delta14-sterol reductase (1.3.1.70), a sterol desaturase (*erg3*), and a lathosterol oxidase (EC 1.14.19.20). They downregulated *hyd1*, which encodes a cholestenol delta isomerase; the cytochrome P450 family member *cyp27b1* (EC 1.14.15.18); and genes encoding a cholestenone-5-alpha-reductase (EC 1.3.1.22), a cholestenol delta-isomerase (EC 5.3.3.5), and a cholesterase (EC 3.1.1.13) (Additional file 7).

### Validation of gene expression resulting from RNAseq by RT-qPCR

**Fig. 6:**
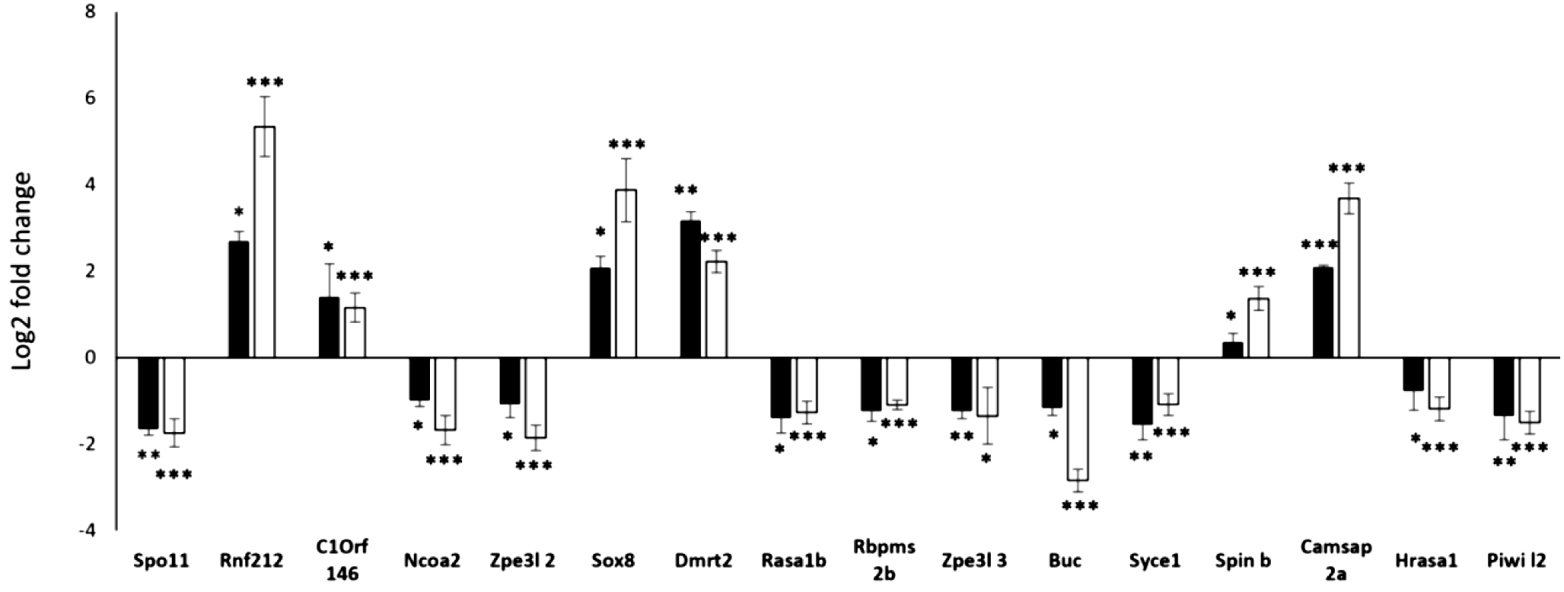
Validation of gene expression resulting from RNAseq by the RT-qPCR approach using 17 reproduction-related genes. The x-axis displays the gene names. The y-axis displays the log2 fold change of the gene expression between sexual females and asexual females of *C. gibelio*. A positive log2 fold change of the gene expression indicates that the gene was upregulated in asexual females when compared to sexual females. A negative log2 fold change indicates that the gene was downregulated in asexual females when compared to sexual females. The data represent the means of five independent biological replicates, and bars represent standard deviation. Asterisks indicate statistically significant differences in the log2 fold change of qPCR data between sexual and asexual females of *C. gibelio* based on Student’s t-test: *p < 0.05, **p < 0.01, ***p < 0.001

To validate the DEGs revealed by RNAseq, we performed RT-qPCR for 17 selected genes involved in reproduction that were significantly up- or downregulated in asexual females of *C. gibelio* compared to sexual females (Table 1). The RT-qPCR analysis confirmed the downregulation of 10 and upregulation of 7 reproduction-associated genes (Fig. 6). There was a positive correlation between the log2 fold change of RNAseq and the log2 fold change of qPCR (*r* = 0.89, *p* < 0.001) (Additional file 8).

## Discussion

The present study analysed the transcriptome profiles of gonadal tissues from *C. gibelio* using RNAseq, specifically to identify DEGs in ovaries associated with reproduction in triploid gynogenetic females and diploid sexual females. We also analysed the transcriptome profiles of gonads in male *C. gibelio*, and males and females of the two closely-related species *C. auratus* and *C. carpio*. A total of 1728 genes were significantly upregulated or downregulated in asexual females of *C. gibelio* compared to sexual females. The transcriptome profiles based on normalized RNAseq read counts showed a sex-dependant difference for both - all transcribed genes or reproduction-associated genes, with an overall similarity between gynogenetic and sexual females of *C. gibelio* and females of *C. auratus*, and an overall similarity between the males of the two *Carassius* species.

GO term overrepresentation analyses and KEGG pathway enrichment analyses indicated an overall overexpression of genes involved in meiosis and cell cycle control (cell cycle, negative regulation of nuclear division, negative regulation of cell cycle process, oocyte meiosis, and synaptonemal complex assembly), oocyte maturation (egg coat formation, structural constituent of egg coat, and calcium ion binding) and fertilization (binding of sperm to zona pellucida, positive regulation of acrosome reaction). Calcium ion binding, which plays critical roles in fertilization and early development (for review, see Whitaker [77]), was also overrepresented in sexual females. This suggests that the regulation of oogenesis, as well as the response of oocytes to sperm cell binding, differ between sexual reproduction and gynogenesis, where the eggs are only activated by the sperm cell (for review, see Schlupp [78]). An overall downregulation of meiotic and reproduction-associated genes was also reported in *Poecilia formosa*, a gynogenetic fish species of the Amazon basin, compared to its sexual parental ancestors, *P. mexicana* and *P. latipinna* [20]. Similar results were reported in invertebrates that use cyclical parthenogenesis, such as the planktonic crustacean *Daphnia*, rotifers, and aphids, where the sexual forms upregulate genes involved in cell cycle control, meiosis, oogenesis, and oocyte maturation [79–82].

On the basis of ovarian transcriptome profiles, we identified around 100 reproduction-associated genes related to oocyte meiosis, oogenesis, embryogenesis, hormone signalling, and fertilization that were differently expressed between sexual and gynogenetic females; the expression pattern of a set of 17 selected genes based on the basis of RNAseq was validated by RT-qPCR. We also specifically analysed 40 meiosis-related genes inferred by previous studies [66–68, 75, 76]. We showed that sexual females upregulated several meiosis-associated genes involved in recombination and crossover and in DNA double-strand break formation during meiosis, including *spo11, msh2*, *pds5b*, *sbk3*, *stag1a*, and *rec114*. Two components of the minichromosome complex (*mcm4* and *mcm9*), involved in crossover inhibition during meiosis [83], as well as *syce1*, a component of the synaptonemal complex that forms between homologous chromosomes during recombination, were also upregulated in sexual females [84–86]. Sexual females also upregulated genes involved in oocyte maturation, such as *emi1* (also named *fbxo5*), a major F-box constituent of the E3 ubiquitin ligase protein that regulates the anaphase promoting complex (APC) during meiosis and mitosis [87–90]; and *spinw*, a major maternal transcript expressed in oocytes during early development. The importance of spindlin in oocytes to embryo transition in *C. gibelio* has been established [91]. Furthermore, several genes involved in cell cycle regulation, including three members of the Ras/MAPK family, *hrasa, hrasb and rasa1b*, which encode GTPases controlling cell growth, division, and differentiation [92–95] through the action of mitogen activated protein kinases [96], were also more expressed in sexual females. This suggests that cell cycle control regulation differs between sexual and gynogenetic females of *C. gibelio*.

In accordance with our results, gynogenetic *P. formosa* was shown to underexpress meiosis-related genes, including *sbk3, setd7* and *stk32c*, compared to its supposed sexual ancestors [20]. Similarly, in cyclically parthenogenetic *Daphnia*, meiosis-related genes, including genes related to the spindle assembly checkpoint, the APC, and meiosis chromosome segregation, were upregulated during sexual reproduction [81]. In particular, *spo11*, which encodes a topoisomerase involved in chromosomal recombination during the meiotic prophase, was also described as an important player in the meiosis-to-parthenogenesis transition in pea aphid [97], although it was not reported in asexual *P. formosa* [20].

However, our study also revealed that meiosis pathways were not fully disrupted in gynogenetic females of *C. gibelio*. They retained detectable expressions of all reproduction-associated genes identified, including meiosis-specific genes, in contrast to *P. formosa*, where some meiosis-related genes were not expressed [20]. According to our analyses, several of the core meiosis specific genes, such as *dmc1, mlh1, mnd1, mre11* and genes of the *msh* family [67, 68, 75, 76], did not show significant differences in expression between sexual and gynogenetic females of gibel carp. Gynogenetic females even upregulated *rad1*, a member of the cell cycle checkpoint, also involved in the recombination process during meiosis; *rnf212*, involved in meiotic recombination; and *mad2l2*, involved in the spindle assembly checkpoint; as well as meiosis-specific genes that were previously found to be downregulated in gynogenetic *P. formosa*, such as *b4galt, clk4, dmrta2, grapb*, and *rasl11b* [20]. However, these results are in accordance with a study suggesting that meiosis is retained even in gynogenetic strains of *C. gibelio* in North-east Asia [98]. Furthermore, meiosis genes were reported not to be necessarily associated with sexual reproduction, since asexual amoeba constitutively expressed meiosis-associated genes [66]. Similar results were reported also in rotifers, where no meiosis-specific genes were differently expressed between parthenogenetic and sexual forms [80], and cyclically-parthenogenetic *Daphnia*, which was shown to express meiosis-specific genes during the parthenogenetic phase [99]. In the pea aphid, several oogenesis and cell cycle-related genes were also upregulated during the asexual reproduction phase [79].

Our results reveal an overall upregulation of pathways related to oocyte maturation in sexual females. They upregulated *buc*, involved in the formation of Balbiani bodies in the oocytes and germ plasm assembly, including follicular epithelium morphogenesis [100]. This gene plays a key role in the specification of oocyte anterior/posterior polarity through interactions with the RNA-binding proteins, such as *rbpms2*, a coactivator of transcriptional activity involved in meiosis and oogenesis [101]. Sexual females of *C. gibelio* also upregulate genes involved in progesterone-mediated oocyte maturation, such as members of the plexin and Wnt families. The Wnt pathway regulator *lbh*, previously reported to be upregulated in females during oocyte maturation in *C. gibeli*o, was also more expressed in sexual females in our study. Similarly, in aphids, genes involved in oocyte axis formation were found to be upregulated during the sexual phase [82]. Furthermore, our analyses support an overall upregulation of sperm-egg recognition and fertilization pathways in sexual females. They upregulated *calm3a*, a member of the calmodulin family responsible for calcium-dependant signal transduction following sperm binding, as well as *plcb4*, a phospholipase involved in oocyte fertilization [102]. In addition, sexual females upregulated components of the zona pellucida, the extracellular matrix surrounding the oocyte involved in sperm-egg recognition [103]. A gene encoding a Ca^2+^-dependant C-type lectin, which was shown to be translocated in cortical granules during oocyte maturation and involved in sperm-egg recognition and fertilization in *C. gibelio* [104], was also significantly upregulated in sexual females. These findings highlight the importance of oocyte maturation, sperm-egg recognition, and fertilization pathways in the coexistence of sexual and asexual females.

Inversely, some genes involved in oocyte development, such as DAZ-like genes, were not differentially expressed between gynogenetic and sexual females of gibel carp in our study, while others, including *bcl2*; the oocyte specific histone *h2af1o*, which plays a key role in fish embryogenesis [105]; and several members of the FGF family, which promote meiosis and maturation of the oocytes [106], were even more expressed in asexual females than in sexual ones. Oocyte maturation and sperm cell binding pathways are not expected to be disrupted in asexual females, since they produce oocytes. Furthermore, gynogenetic *C. gibelio* females still require sperm cell binding to activate the eggs [78, 107]. The overexpression of some oogenesis-related genes was also reported in aphids during the parthenogenetic phase of their life cycle [79]. Furthermore, the downregulation of *uhrf1*, an oocyte-specific epigenetic regulator [108] in sexual females of *C. gibelio*, also reported in aphids [79], suggests a difference in the epigenetic regulation of oogenesis between sexual and asexual forms. Hence, these results suggest that many genes and pathways are involved in both parthenogenetic oogenesis and sexual oogenesis in *C. gibelio*. However, gene expression differs between the two reproduction forms. It is noteworthy that members of the same gene family can be up- or downregulated, such as members of the zona pellucida and F-box families. Such divergent expression, also reported in *Daphnia* [81], may suggest functional divergence among members of the same multigenic families.

Our analyses also suggest differences in hormonal signalling and sex differentiation processes between sexual and gynogenetic reproduction. Components of the GnRH signalling pathway, and genes linked to ovarian fertility, such as the gene encoding the luteinizing hormone/choriogonadotropin receptor *(lhcgr)*, were more expressed in sexual females. The TGF-β signalling pathway, involved in many physiological processes including sexual differentiation in fish [109–111], was also differently regulated between gynogenetic and sexual females of *C. gibelio*. Sexual females upregulated *smad* genes, involved in oogenesis, ovarian function, and folliculogenesis *via* the negative regulation of TGF-β signalling [108, 112–114]. Regarding gynogenetic females, they upregulated two *dmrt* genes. These genes were shown to promote male differentiation and repress female-specific differentiation of the gonads, and they are also involved in brain sexual differentiation [114–118] as well as in XY reversal in sex-alternating fish species [115]. Gynogenetic females of *C. gibelio* also upregulated *ncoa2*, a transcriptional coactivator of steroid receptors and nuclear receptor, as well as *sox8*, involved in female sex determination [119], meiotic progression, and embryonic development [120], and inhibin alpha (*inha)*, involved in steroid hormone biosynthesis. Ovarian aromatase or estrogen synthetase (*cyp19a1a*), a member of the cytochrome P450 subfamily involved in steroidogenesis [121] and female folliculogenesis and gonadal differentiation, was also upregulated in gynogenetic females of *C. gibelio*, as was *oxtr*, a gene encoding the oxytocin receptor, a component of the oxytocin signalling system that modulates reproductive behaviour. Our results also suggest that sexual females upregulated some genes associated with the steroid hormone synthesis pathway. The hydroxysteroid 17-β-dehydrogenase gene *hsd17b1*, which is both estrogenic [122] and androgenic [123], was more expressed in gynogenetic females. Furthermore, sexual females also upregulated the germ cell maintenance gene *piwil2*, a member of the Argonaute family involved in male fertility [124].

In this study, we also investigated the evolutionary history of *C. gibelio*. Ploidy changes shaped the evolution of cyprinids, particularly that of the *Carassius auratus* complex. This complex was formed by allotetraploidization [42, 125] and further polyploidization events have been reported in diverse lineages of the complex, including *C. auratus* and *C. gibelio* [42, 126]. The evolutionary origin of *C. gibelio* is still in question. A study based on *dmrt* genes suggested a recent autopolyploidization event within the *C. auratus* complex that generated the triploid gynogenetic *C. gibelio* [41]. However, an origin of *C. gibelio* by hybridization between *C. auratus* and *C. carpio* has also been proposed [45]. Our SNP clustering, based on gonadal transcriptomes, using *C. gibelio*, *C. auratus* and *C. carpio*, suggests a close evolutionary relationship between sexual and gynogenetic *C. gibelio*, as well as a close relatedness between *C. gibelio* and *C. auratus*, even though we identified SNPs shared only by *C. gibelio* and *C. carpio*, suggesting some genetic contribution of *C. carpio* to the genome of *C. gibelio*. The study of Yuan et al. [118] proposed that triploid gynogenetic *C. gibelio* (3n = 150) resulted from interspecific hybridization between diploid *C. auratus* (2n = 100) and *C. carpio* (2n = 100), contributing with two sets and one set of chromosomes, respectively. Specifically, their study showed that two gene copies of four different *Hox* genes in the genome of gynogenetic *C. gibelio* are orthologous to the *Hox* genes of *C. auratus* and that one is orthologous to the *Hox* gene of *C. carpio* [45]. However, the diploid form of *C. gibelio* was not included in that study. Other studies using mtDNA and *hoxa2b* gene sequences even suggested a more complex relationship between *C. gibelio* and *C. auratus*, where the monophyly of *C. gibelio* was not supported [127, 128]. In addition, gene flow was highlighted between the two species [98, 127], suggesting that *C. gibelio* and *C. auratus* were conspecific and interfertile.

Ploidy changes often affect meiosis, and parthenogenetic species usually result from interspecific hybridization [8] with some exceptions [129]. Polyploidy can lead to the formation of unreduced eggs whose cell cycle is arrested at the metaphase of meiosis II [130]. This results in asexually reproducing species, where the offspring are clones of the mother. Unisexual fish reproduce through gynogenesis, where the sperm from males of the same or closely-related species is still required to activate the egg. Still, because meiosis pathways were not disrupted, a later genetic contribution from a sperm donor such as *C. auratus* and *C. carpio* cannot be excluded. Such a case of a complex evolutionary history was reported in the unisexual salamander *Ambystoma*. However, in this case, the haploid genome of the sperm donor replaced the nuclear genome, a phenomenon known as kleptogenesis [131, 132].

Our results suggest that all along their evolutionary history, asexual lines of *C. gibelio* did not lose the genetic toolkit for meiosis, and that the sexual reproduction genetic toolkit is not under relaxed selection, a condition also reported in asexual *P. formosa* [20] and snails [133]. The re-acquisition of sexual reproduction in asexual species is very rare and very few cases have been reported. Either some gynogenetic *C. gibelio* females were able to secondarily regain sexual reproduction and to produce both diploid and triploid males, or a minority of sexual individuals still persisted within the already formed gynogenetic form and became more abundant later [68]. In all cases, this led to the current sympatric coexistence of sexual and gynogenetic individuals [27, 32]. Polyploidy in general, and triploidy in the case of gynogenetic *C. gibelio* could possibly compensate the deleterious effects of Muller’s ratchet or the accumulation of deleterious mutations by increasing the number of gene copies and favouring heterozygosity [66]. The genomic incorporation of sperm-derived fragments from an exogenous species, which was reported in gynogenetic *C. gibelio* from aquaculture in China [33], can also favor genetic diversity in asexual lines. In *C. gibelio*, the combination of the advantages of gynogenetic reproduction, which allows for faster population growth [26], and sexual reproduction, which provides higher resistance to parasites and higher immune gene variability [28], higher aerobic performance and better immunity [134], lower metabolic rate, and lower energy intake [135], might explain the coexistence of sexual and asexual forms, and the high adaptive abilities of this species and its invasiveness in European water ecosystems.

### Electronic supplementary material

Below is the link to the electronic supplementary material.

Supplementary Material 1

Supplementary Material 2

Supplementary Material 3

Supplementary Material 4

Supplementary Material 5

Supplementary Material 6

Supplementary Material 7

Supplementary Material 8

## Data Availability

The data used in this study have been deposited in NCBI´s Gene Expression Omnibus and are accessible through GEO Series accession number GSE254010 (https://www.ncbi.nlm.nih.gov/geo/query/acc.cgi?acc=GSE254010).
