# Supplementary material for: Reproduction-associated pathways in females of gibel carp (Carassius gibelio) shed light on the molecular mechanisms of the coexistence of asexual and sexual reproduction
Source: BMC Genomics. 2024 Jun 1;25:548. doi: 10.1186/s12864-024-10462-4 (PMC11144346; doi:10.1186/s12864-024-10462-4)
Supplement: Supplementary file 1 — Supplementary Material 1 [file 12864_2024_10462_MOESM1_ESM.docx]

**Additional file 1**: Number and percentage of uniquely- and multi-mapped reads, and percent of multimapped reads per sample. Ca: *Carassius auratus*. Cc: *Cyprinus carpio*. Cg: *Carassius gibelio*. Legend: rep: replicate, F: female, M: male.

| Sample | uniquely mapped | uniquely mapped (%) | multimapped | multimapped (%) |
| --- | --- | --- | --- | --- |
| Ca_2n_f_rep1 | 11,187,375 | 78.01 | 2,068,965 | 14.43 |
| Ca_2n_f_rep2 | 8,395,893 | 72.64 | 1,827,930 | 15.81 |
| Ca_2n_f_rep3 | 9,157,357 | 71.98 | 2,032,446 | 15.97 |
| Ca_2n_f_rep4 | 8,469,867 | 72.20 | 2,003,179 | 17.08 |
| Ca_2n_f_rep5 | 7,642,191 | 65.49 | 2,292,890 | 19.65 |
| Ca_2n_m_rep1 | 10,158,435 | 83.52 | 1,502,949 | 12.36 |
| Ca_2n_m_rep2 | 10,119,793 | 83.79 | 1,490,306 | 12.34 |
| Ca_2n_m_rep3 | 8,570,927 | 82.41 | 1,356,860 | 13.05 |
| Ca_2n_m_rep4 | 11,797,446 | 82.78 | 1,824,108 | 12.80 |
| Ca_2n_m_rep5 | 9,781,738 | 82.84 | 1,447,812 | 12.26 |
| Cc_2n_f_rep1 | 4,911,330 | 53.72 | 1,227,847 | 13.43 |
| Cc_2n_f_rep2 | 6,365,520 | 51.18 | 1,987,990 | 15.98 |
| Cc_2n_f_rep3 | 6,192,931 | 54.08 | 1,593,646 | 13.92 |
| Cc_2n_f_rep4 | 7,498,160 | 55.75 | 1,801,259 | 13.39 |
| Cc_2n_m_rep1 | 6,266,243 | 68.10 | 1,140,262 | 12.39 |
| Cc_2n_m_rep2 | 8,835,887 | 64.96 | 1,607,576 | 11.82 |
| Cc_2n_m_rep3 | 4,748,924 | 64.89 | 878,044 | 12.00 |
| Cc_2n_m_rep4 | 7,250,330 | 63.52 | 1,422,296 | 12.46 |
| Cg_2n_f_rep1 | 4,790,628 | 63.42 | 1,560,019 | 20.65 |
| Cg_2n_f_rep2 | 5,786,200 | 59.84 | 2,149,729 | 22.23 |
| Cg_2n_f_rep3 | 8,581,687 | 63.10 | 2,732,608 | 20.09 |
| Cg_2n_f_rep4 | 8,029,779 | 62.91 | 2,600,361 | 20.37 |
| Cg_2n_f_rep5 | 4,827,139 | 63.50 | 1,524,954 | 20.06 |
| Cg_2n_m_rep1 | 12,316,093 | 82.05 | 2,004,938 | 13.36 |
| Cg_2n_m_rep2 | 10,386,825 | 63.48 | 3,317,699 | 20.27 |
| Cg_2n_m_rep3 | 9,414,396 | 83.27 | 1,431,373 | 12.66 |
| Cg_2n_m_rep4 | 11,028,319 | 82.46 | 1,826,441 | 13.66 |
| Cg_2n_m_rep5 | 10,904,789 | 82.35 | 1,765,222 | 13.33 |
| Cg_3n_f_rep1 | 7,284,881 | 61.43 | 2,519,547 | 21.25 |
| Cg_3n_f_rep2 | 9,295,633 | 62.59 | 3,033,749 | 20.43 |
| Cg_3n_f_rep3 | 5,370,617 | 62.02 | 1,798,414 | 20.77 |
| Cg_3n_f_rep4 | 10,120,589 | 60.85 | 3,633,352 | 21.85 |
| Cg_3n_f_rep5 | 7,633,902 | 62.12 | 2,599,037 | 21.15 |
| Cg_3n_m_rep1 | 9,637,948 | 79.76 | 1,840,115 | 15.23 |
| Cg_3n_m_rep2 | 11,202,537 | 82.52 | 1,807,278 | 13.31 |
| Cg_3n_m_rep3 | 10,633,698 | 82.13 | 1,764,095 | 13.63 |
| Cg_3n_m_rep4 | 11,273,455 | 79.99 | 2,100,484 | 14.90 |
| Cg_3n_m_rep5 | 12,317,538 | 81.85 | 2,116,720 | 14.06 |
