# Supplementary material for: Reproduction-associated pathways in females of gibel carp (Carassius gibelio) shed light on the molecular mechanisms of the coexistence of asexual and sexual reproduction
Source: BMC Genomics. 2024 Jun 1;25:548. doi: 10.1186/s12864-024-10462-4 (PMC11144346; doi:10.1186/s12864-024-10462-4)
Supplement: Supplementary file 3 — Supplementary Material 3 [file 12864_2024_10462_MOESM3_ESM.docx]

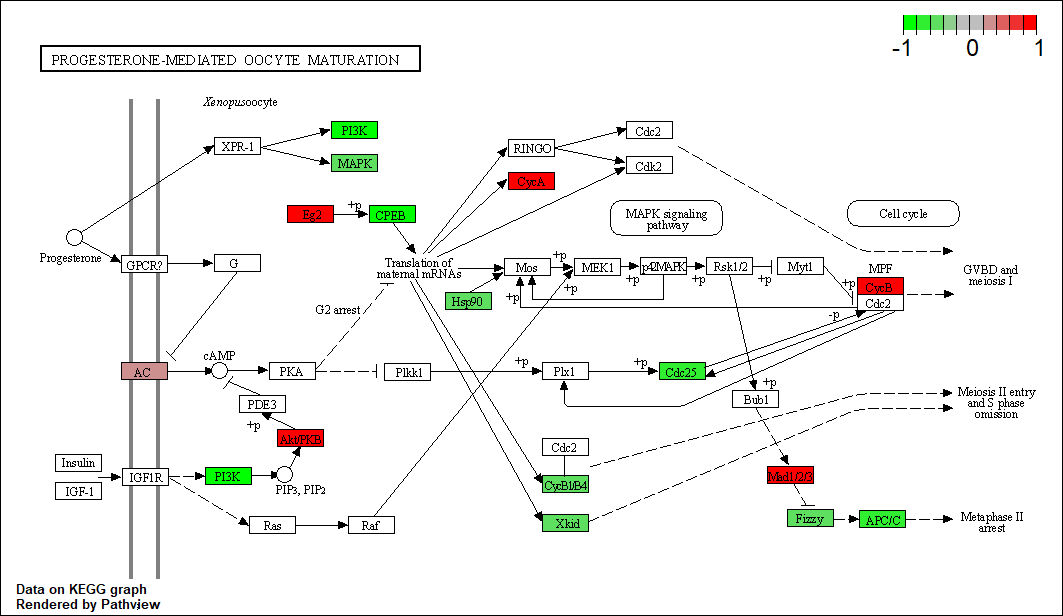


**Additional file 4**: KEGG pathway map of progesterone-mediated oocyte maturation. Colors indicate the significant upregulation (red) or downregulation (green) of the genes in gynogenetic females compared to sexual ones.
