# Supplementary material for: Reproduction-associated pathways in females of gibel carp (Carassius gibelio) shed light on the molecular mechanisms of the coexistence of asexual and sexual reproduction
Source: BMC Genomics. 2024 Jun 1;25:548. doi: 10.1186/s12864-024-10462-4 (PMC11144346; doi:10.1186/s12864-024-10462-4)
Supplement: Supplementary file 7 — Supplementary Material 7 [file 12864_2024_10462_MOESM7_ESM.docx]

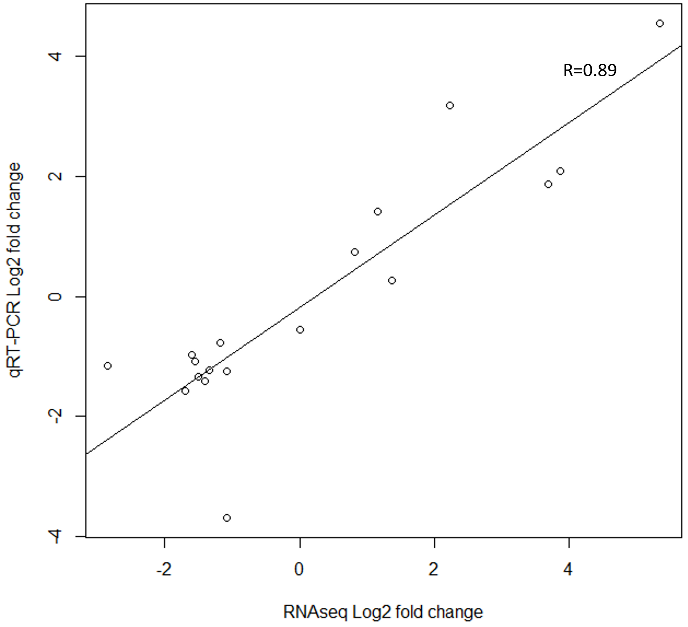


**Additional file 8**: Correlation between log2 fold change in the gene expression from RNAseq and RT-qPCR of the selected 17 reproduction-associated genes. Pearson’s correlation coefficient (R) is shown.
