# Supplementary material for: Reproduction-associated pathways in females of gibel carp (Carassius gibelio) shed light on the molecular mechanisms of the coexistence of asexual and sexual reproduction
Source: BMC Genomics. 2024 Jun 1;25:548. doi: 10.1186/s12864-024-10462-4 (PMC11144346; doi:10.1186/s12864-024-10462-4)
Supplement: Supplementary file 8 — Supplementary Material 8 [file 12864_2024_10462_MOESM8_ESM.docx]

**A**
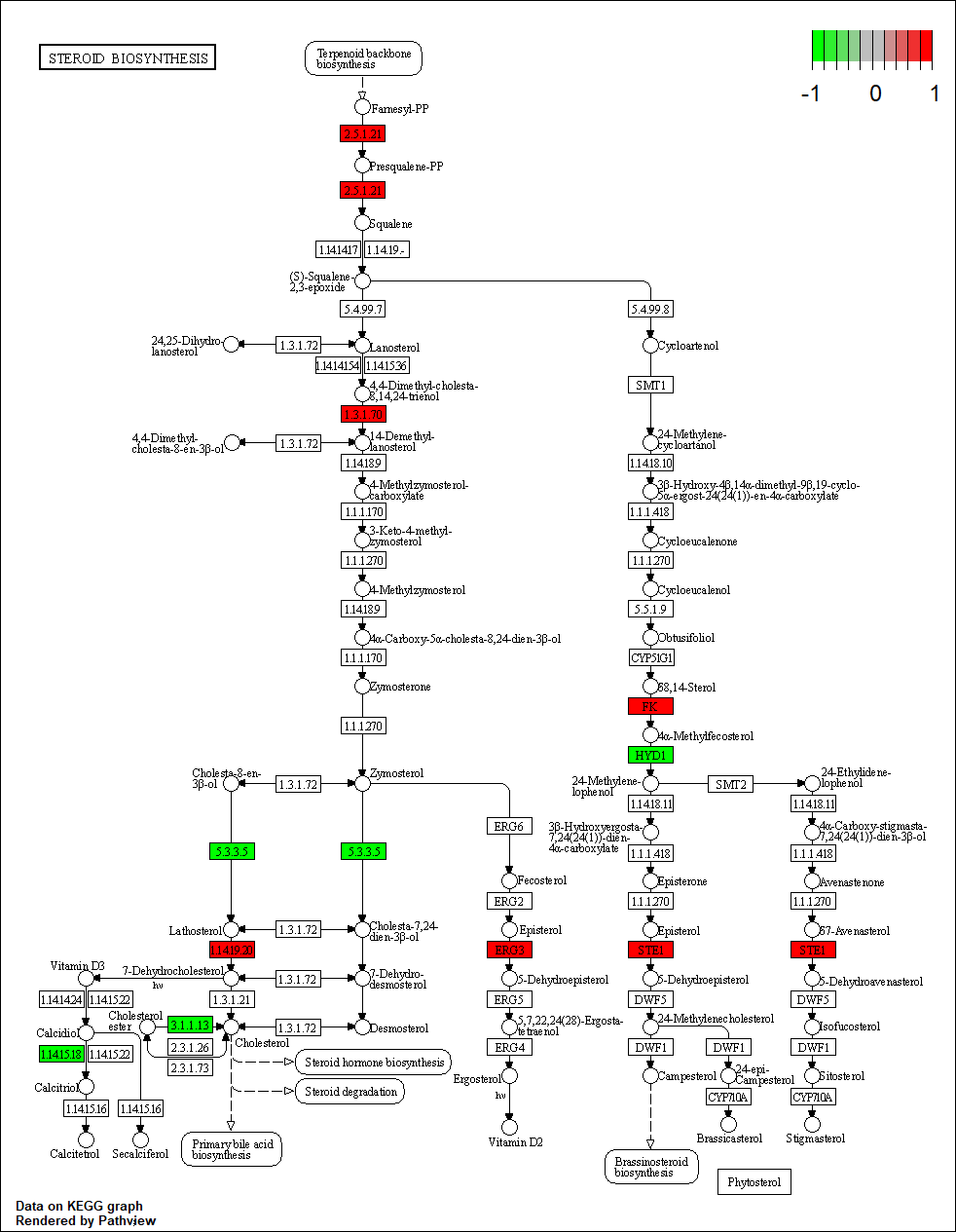


**B**
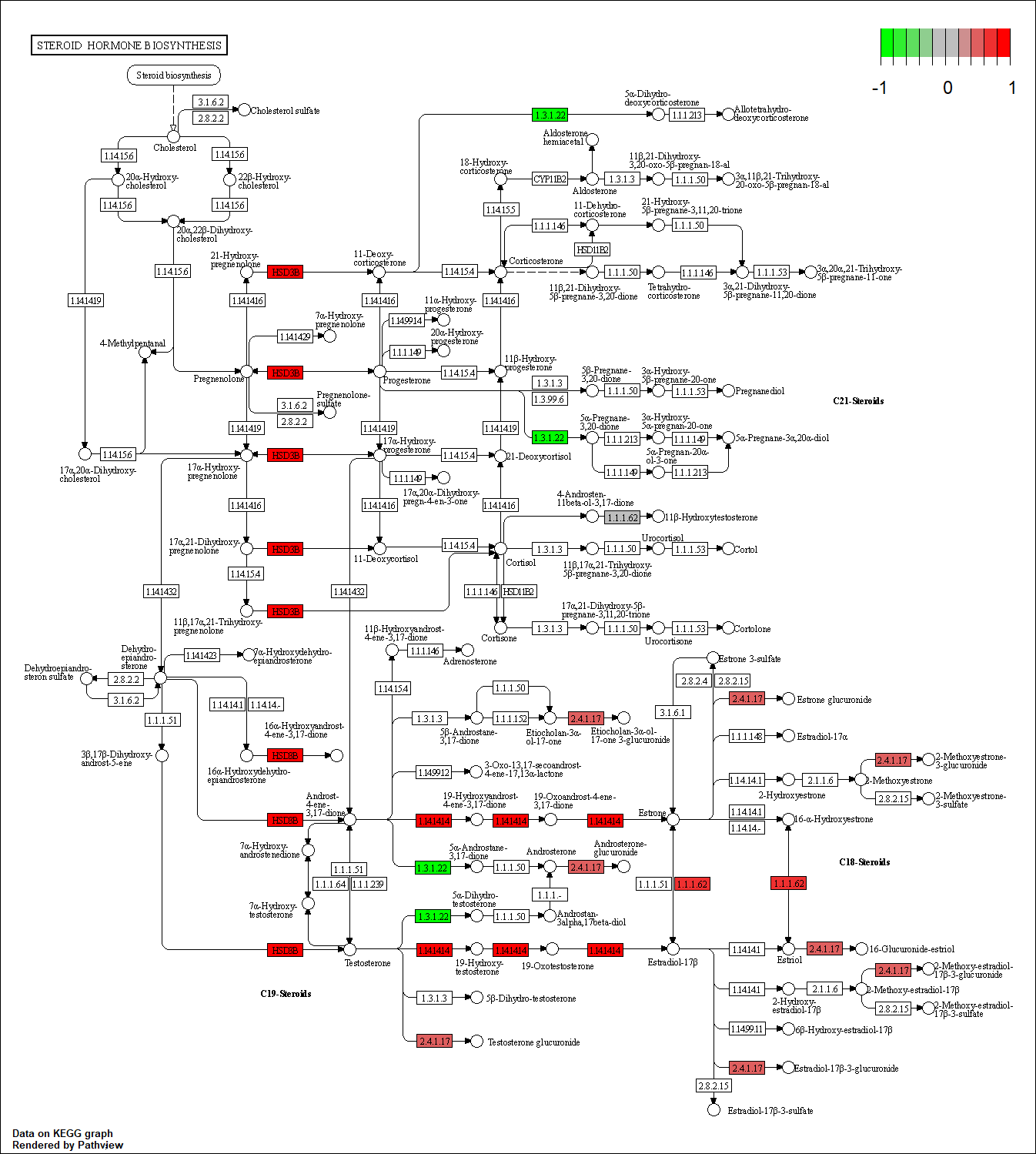


**Additional file 7**: (A) KEGG pathway map of steroid biosynthesis (A) and steroid hormone biosynthesis (B). Colors indicate the significant upregulation (red) or downregulation (green) of the genes in gynogenetic females compared to sexual ones.
